# Optimization of SARS-CoV-2 Pseudovirion Production in Lentivirus Backbone With a Novel Liposomal System

**DOI:** 10.3389/fphar.2022.840727

**Published:** 2022-03-25

**Authors:** Gokulnath Mahalingam, Hari Krishnareddy Rachamalla, Porkizhi Arjunan, Yogapriya Periyasami, Salma M, Saravanabhavan Thangavel, Kumarasamypet M. Mohankumar, Mahesh Moorthy, Shaji R. Velayudhan, Alok Srivastava, Srujan Marepally

**Affiliations:** ^1^ Centre for Stem Cell Research (CSCR) (a Unit of InStem, Bengaluru), CMC Campus, Vellore, India; ^2^ CSIR-Indian Institute of Chemical Technology, Hyderabad, India; ^3^ Department of Clinical Virology, Christian Medical College, Vellore, India

**Keywords:** liposomes, pseudoviral neutralization, SARS-CoV-2 (COVID-19), transfection, lentivirus

## Abstract

Due to the fast mutating nature of severe acute respiratory syndrome coronavirus 2 (SARS-CoV-2), the development of novel therapeutics, vaccines, and evaluating the efficacies of existing one’s against the mutated strains is critical for containing the virus. Pseudotyped SARS-CoV-2 viruses are proven to be instrumental in evaluating the efficiencies of therapeutics, owing to their ease in application and safety when compared to handling the live virus. However, a comprehensive protocol that includes selecting transfection reagents, validating different packaging systems for high-throughput screening of neutralizing antibodies, is still a requisite. To this end, we designed and synthesized amide linker-based cationic lipids with varying hydrophilic head groups from dimethyl (Lipo-DME) to methyl, ethylhydroxyl (Lipo-MeOH), and diethylhydroxyl (Lipo-DOH) keeping the hydrophobic tail, stearic acid, as constant. Among the liposomal formulations of these lipids, Lipo-DOH was found to be superior in delivering plasmids and demonstrated comparable transfection efficiencies with commercial standard Lipofectamine 3000. We further used Lipo-DOH for lentivirus and SARS-CoV-2 pseudovirion preparation. For comparing different lentivirus packaging systems, we optimized conditions using Addgene and BEI systems and found that the BEI lenti plasmid system was found to be efficient in making lentiviruses using Lipo-DOH. Using the optimized transfection reagent and the lentivirus system, we developed a robust protocol for the generation of SARS-CoV-2 pseudovirions and characterized their infectivity in human ACE2 expressing HEK-293T cells and neutralizing properties in IgG against spike protein of SARS-CoV-2 positive human sera from individuals recovered from COVID-19.

## Introduction

The world has been going through a traumatic phase caused by severe acute respiratory syndrome coronavirus 2 (SARS-CoV-2) and its variants in the coronavirus disease (COVID-19) pandemic (Dhar et al., 2021; Farooqi et al., 2021; Tao et al., 2021). The spike (S) glycoprotein present on the virus envelope is responsible for attachment and entry into host cells by binding to human angiotensin-converting enzyme 2 (hACE2) receptors (Hoffmann et al., 2020b). The vaccines available against SARS-CoV-2 including mRNA, protein subunit, and viral vaccines induce a robust immune response resulting in the production of neutralizing antibodies (nAbs) that efficiently neutralize virus infectivity, thereby providing in virus neutralization which correlated with protection from re-infection or COVID-19 (Corbett et al., 2020; Erasmus et al., 2020; Laczkó et al., 2020; De Alwis et al., 2021; Kyriakidis et al., 2021; Mandolesi et al., 2021; Martínez-Flores et al., 2021). The level of nAbs is an important parameter to determine the disease progression, quality of convalescent plasma (which has been used to treat hospitalized patients to prevent COVID-19 progression), and vaccines efficacy. (Zeng et al., 2020; Chmielewska et al., 2021; Klassen et al., 2021). Recently reported that the SARS-CoV-2 variants, especially new variants of concern (VOCs) increase disease transmissibility and mortality than wild SARS-CoV-2 type (Chmielewska et al., 2021; Harvey et al., 2021). Thus, it necessitates the development of high-throughput and low-cost methods for screening of nAbs in the COVID-19 pandemic for the development of novel therapeutic strategies. The assays are widely adopted for determining the level of nAbs by using a live virus (such as micro-neutralization (MN) and the plaque reduction neutralization test (PRNT)) and replication-deficient pseudovirions expressing SARS-CoV-2 surface proteins. Handling of the live virus requires handling with biosafety level 3 (BSL-3) practices, thus limiting its use to only in facilities with higher containment. MN and PRNT are low-throughput assays and cannot be deployed to scale for population-based testing. As SARS-CoV-2 is highly infective and pathogenic in nature, the live virus is extremely challenging, has low-throughput, and requires BSL-3 (Bewley et al., 2021; Lu et al., 2021).

Pseudotyped SARS-CoV-2 viruses have been instrumental in evaluating the efficiencies of nAbs, as the pseudovirus manufacturing and handling requires a less stringent BSL-2 facility (Crawford et al., 2020; Lu et al., 2021). Hence, their applications were well popularized in the pandemic period. To mimic the natural infectivity of the virus, recent protocols have been used for the production of pseudoviruses expressing spike glycoprotein of SARS-CoV-2 in other viral backbones such as murine leukemia virus (MLV), vesicular stomatitis virus (VSV), and human immunodeficiency virus (HIV) (Almahboub et al., 2020; Condor Capcha et al., 2020; Crawford et al., 2020; Hyseni et al., 2020; Johnson et al., 2020; Nie et al., 2020; Schmidt et al., 2020; Donofrio et al., 2021; Kalkeri et al., 2021; Lu et al., 2021; Neerukonda et al., 2021). The efficiency of pseudovirus production critically depends on the backbone used in making the pseudovirus and the efficiency of the transfection reagent in delivering the viral plasmids into the cells. To the best of our knowledge, a comprehensive manufacturing protocol for SARS-CoV-2 pseudovirus that includes selecting transfection reagents and validating different packaging systems for facile and robust manufacturing is not reported yet.

Among the viral backbones, the human immunodeficiency virus (HIV) backbone and the lentivirus system have been used widely in gene transfer applications. Lentiviruses have vesicular stromatitis virus glycoprotein (VSV-G) on their envelope for broader infectivity into multiple cell types (Connolly, 2002). Typically, the lentivirus system contains packaging plasmid, envelope plasmid, and transfer plasmids. Sequences of spike protein were inserted in the place of VSV-G in the case of the SARS-CoV-2 pseudovirus system (Wu et al., 2000). Usually, these plasmids are transfected with the help of transfecting reagents into HEK-293T for producing viruses. Polyetheneimine (PEI) is the most commonly used transfection reagent (Yang et al., 2017). However, high cytotoxicity and a requisite for media change post-transfection result in low titer virus production (Corbett et al., 2020). Other commercially available liposomal transfection reagents, such as Lipofectamine 3000, are efficient with low cytotoxicity that results in high titer production (Lana and Strauss, 2020). However, the high cost of the transfection reagent is a challenge for large scale pseudovirus production for high-throughput screening (Martínez-Molina et al., 2020).

Liposomal transfection involves three critical steps including complete complexation with pDNA at lower lipid to DNA charge ratio, effective cellular uptake, and early escape from endosome that determine their transfection efficiencies (Dharmalingam et al., 2017). Typically, lipid contains a hydrophilic head group, hydrophobic tail, and linker functionality that connect both the hydrophilic head group and the hydrophobic tail (Chandrashekhar et al., 2011). In our previous studies, we conducted a structure activity investigation on the molecular architecture of the lipids by varying the hydrophilic head group, hydrophobic tail, and linker functionality and demonstrated that a cationic lipid with amide linker functionality was found to be efficient in delivering nucleic acids into multiple cell lines (Srujan et al., 2011; Andey et al., 2014). For improving the transfection efficiencies further, we designed synthesized amide linker-based cationic lipids by varying their hydrophilic domain from dimethyl to methyl, hydroxyethyl, and dihydroxyethyl and evaluated their transfection efficiencies for choosing a transfection reagent for lentivirus production.

In this study, we demonstrated detailed procedures for making a less expensive in-house transfection reagent, selected best HIV backbone for making SARS-CoV-2 spike pseudovirus, and evaluated neutralization assay using a commercial spike antibody, soluble hACE2, and human convalescent sera.

## Materials and Methods

### Synthesis of Amide Linker-Based Cationic Lipids

Complete experimental details including the syntheses, spectral characterization (^1^H-NMR and ESI-MS for intermediates and ^1^H-NMR, ^13^C-NMR, ESI-MS, and HRMS for target molecules) of the cationic lipids, and their corresponding intermediates are provided in the supplementary information. The purities of all the final cationic lipids were evaluated by HPLC (high-performance liquid chromatography).

### Preparation of Liposomes

Liposomes (1 mM) of DMe18, MeOH18, and DOH18 were prepared with 1:1:1 mol ratios of each lipid, co-lipids DOPE (1,2-dioleoyl-sn-glycero-3-phosphoethanolamine), and cholesterol using the established protocol in the lab (Srujan et al., 2011). In brief, lipid stocks were mixed at the desired molar ratio (1:1:1) in a glass vial, and it formed a thin film under the flow of moisture-free nitrogen gas and dried under vacuum for 6 h. The thin lipid films were hydrated in 1 ml of deionized water overnight (approx. 8 h). Post-hydration, the formulations were subjected to vortex form 2–3 min at room temperature, followed by probe sonication using a Branson 450 Sonifier at 100% duty cycle and 25 W output power to produce small unilamellar vesicles.

### Particles Zeta Potentials (ξ) and Hydrodynamic Diameter Measurements

Hydrodynamic diameters and the surface potentials of the cationic liposomes, that is, Lipo-DME, Lipo-MeOH, and Lipo-DOH were measured in a Litesizer™ 500 Particle Analyzer (Anton Paar, Austria). The diameter of the particles was analyzed in Milli-Q water. The system was calibrated by using the 200 nm + 5 nm polystyrene polymer (Duke Scientific Corps. Palo Alto, CA). The zeta potential was measured using the following parameters: viscosity, 0.89 cP; dielectric constant, 79; temperature, 25°C; F(Ka), 1.50 (Smoluchowski); maximum voltage of the current, V. Measurements were done ten times with the zero-field correction. The particles surface charges were measured by using the Smoluchowski approximation.

### TEM Analysis of Nanoformulations

High-resolution transmission electron microscopic (HR-TEM) images of the liposomes were captured using a JEOL-JEM 2100. Fleetingly, 5 μL of 1 mM nanoformulations solution was positioned on a 200-mesh carbon-coated copper grid (glow discharged for 45 s using the Tolaron Hivac Evaporator) and kept for 10 minutes. An additional sample was removed by using a filter paper. In addition, 5 μL of 2% uranyl acetate polymeric solution was added to the abovementioned carbon-coated copper grid, kept for 2 minutes, air-dried, and measured at 120 KV.

### pDNA Binding Assay

The ability of liposome complexation was assessed by a gel retardation assay on a respective percentage of agarose gel. The lipid was taken in varying charge ratios with a constant DNA concentration ranging from 1:1 to 4:1. Liposome was complexed with plasmid DNA in a total volume of 40 µL in nuclease-free water and incubated for 30 min at room temperature in a shaker with low speed. The lipoplex was mixed with 6X loading dye, and the complexion efficiency was confirmed by pre-stained ethidium bromide agarose gel.

### DNase I Sensitivity Assay

In the lipoplex (lipid: mRNA) complex, DNase 1X buffer and 0.5 unit of DNase I was added. Then we gently tapped the samples and incubated the mixture for 30 min at room temperature. After incubation, 5 µg of proteinase K was added and kept in a thermomixer at 50°C for 15 min. Finally, 100 µL of phenol:chloroform was added, shaken thoroughly, and incubated for 5 min at room temperature. We centrifuged the samples at 14,000 rpm for 15 min and collected the aqueous layer and confirmed liposome’s efficacy against DNase I enzyme by agarose gel electrophoresis.

### Cytotoxicity Assay

The cytotoxic influence of lipoplex on HEK-293T cell lines was analyzed using MTT (3-(4,5-dimethylthiazol-2-yl)-2, 5-diphenyltetrazolium bromide)-based cytotoxicity assays at a varying charge ratio of 1:1 to 4:1 of lipid:DNA of cationic lipid DOH18, MeOH18, and DME18 in 96-well plates with 80%–90% of cell confluency. After 48 h, MTT (0.5 mg/ml in DMEM) was added to cells and incubated for 4 h at 37°C. Then the reaction was stopped by adding 200 µL of DMSO, and absorbance was taken at 550 nm. Results were calculated as percent viability = [A540 (treated cells) − background/A540 (untreated cells) − background] x 100.

### *In vitro* Transfection

HEK-293T cells were seeded at a density of 45,000 cells per well in a 48-well plate 18–24 h before the transfection. Then 0.4 μg of plasmid DNA (BEI #-52516) was complexed with diverse concentrations of cationic lipids ranging in the ratio of 1:1 to 4:1 (liposomes:DNA) in a serum-free medium that was made up to 100 μL and incubated for 30 min at room temperature. After the lipoplex formation, the mixture was added to the cells. At 48-h post-transfection, the cationic liposome transfection efficiency was validated by flow cytometry quantitatively and imaging qualitatively.

### hACE2 Stable HEK-293T (293T-hACE2) Cell Line Generation

VSV-G lentiviral particles expressing hACE-P2A-Puro gene were produced by transfecting pLenti-hACE-P2A-Puro using second generation lentiviral plasmids (Addgene) with LF-3000. The lentiviral particles were purified and transduced to 0.5 million HEK-293T cells with 2 ml of D10 media consisting of 10 µL of 1 M HEPES buffer and two polybrene (6 mg/ml) in a six-well plate. At 48-h post transduction, hACE2 stable clones were selected by using puromycin at 2 μg/ml concentration in the D10 medium. The expression of hACE2 clones was confirmed by anti-ACE2 antibodies staining followed by a flow cytometry analysis.

### Production of Pseudotyped Lentiviruses

The pseudotyped lentiviruses were produced as described in Crawford et al. (2020). In brief, 5 × 10^5^ HEK-293T cells were seeded on a six-well plate with 2 ml of D10 growth media (10% FBS in DMEM media). After 18–24 h (at 50–70% confluent), cells were transfected with lentiviral plasmids from Addgene and BEI using Lipo-DOH and LF-3000 formulation, as given in Supplementary Table S1. At 18–24 h post-transfection, 2 ml of fresh D10 media was changed and incubated at 37°C for 60 h. Later, pseudoviruses were harvested by collecting the supernatant from each well, filtered by using a 0.45 µm PVDF low protein-binding filter, and stored at −80°C for further analysis.

### P24 ELISA

The level of p24 core protein in each pseudoviral supernatant was quantified using the DuoSet^®^ ELISA kit (R&D System). Each sample was diluted (1/10) in D10 media with 0.5% Triton X-100 and incubated at 37°C for 30 min. Later, we performed p24 ELISA and quantified as per the manufacturing protocol.

### Pseudotyped Lentiviral Titer

The functional titer of pseudotyped lentiviral particles was determined by flow cytometry and a luciferase assay. In brief, the HEK-hACE2 cells were seeded (1.25 × 10^4^ per well) in 0.1 mg/ml poly-l-lysine-coated 96-well plate. After 12–24 h, we counted the cells in two well to determine the exact cell number for infectivity and transduced with different dilutions of each pseudotyped lentiviral supernatant in D10 media in a final volume of 100 µL. For over expression of hTmprss2 proteins, HEK-hACE2 cells were transfected with pTmprss2 plasmids (Addgene # 53887) 12 h prior to the pseuvirus transduction. At 48–72 h post-transduction, cells were imaged using a fluorescent microscope, followed by an analysis in flow cytometry and the luciferase assay.

### Transduction Unit (TU) Quantification by Flow Cytometry

At post-transduction, cells were harvested by trypsin and resuspended in PBS. The ZsGreen positive cells were quantified by BD Celesta in the FITC channel, and we calculated the TU per ml by the following formula: [Number of cells transduced x (% of ZsGreen positive cells/100) x dilution factor)/(transduction volume (mL))]

### Luciferase Assay

Removed the media at 72 h post transductions from wells and added 50 µL of fresh D10 media to each well. Later, we added 50 µL of Steady-Glo^®^ luciferase reagent for cell lysis and incubated at 5 min. The lysates were transferred to block the 96-well plate and measure the luminescence at 1 s integration time in an i3X plate reader. The RLU per ml was calculated and plotted on the graph.

### Competitive ELISA

The high-affinity spike specific antibodies were estimated in human COVID-19 sera by ACE2 competition ELISA. In brief, we incubated 50 µL of 10-fold serially diluted serum samples in a dilution buffer with 50 µL of biotinylated hACE2 protein in the ELISA plate which was coated with trimeric SARS-CoV-2 S protein. For negative control (NC) and positive control (PC), we added 100 µL dilution buffer and 100 µL of dilution buffer and biotinylated hACE2 protein (1:1), respectively. Later, the plate was washed thrice and incubated with streptavidin-HRP solution for detection of spike-bounded biotinylated hACE2 in each well. The percentage of hACE2–spike interaction was calculated by the following formula: [OD of sample–OD of NC)/(OD of PC–OD of NC)] × 100%.

### Spike-Pseudovirus Neutralization Assay

Spike-pseudoviruses were generated using Lipo-DOH liposome which was used to perform the neutralization test using an anti-RBD antibody, soluble hACE2, and human COVID-19 sera. The 50 µL serially diluted samples in D10 media were mixed with 50 µL of spike-pseudoviral supernatant and kept at 37°C for 1 h in a CO_2_ incubator. A total of 100 µL D10 media and 100 µL of spike-pseudoviral supernatant and D10 media (1:1) served as negative (NC) and positive control (PC), respectively. Later, the neutralized spike-pseudoviruses were transduced with 1.25 × 10^4^ HEK-hACE2 cells in poly-l-lysine-coated 96-well plate. At 72 h post transduction, the luciferase assay was performed as mentioned before, and we calculated the percentage of infectivity using the following formula: [RLU of sample–RLU of NC)/(RLU of PC–RLU of NC)] × 100%.

### qPCR Analysis

Total RNA isolated from HEK-293T and HEK-hACE2 cells were transfected with the pTmprss2 plasmid by the TRIzol method. A total of 500 ng of total RNAs are converted into cDNA using a cDNA synthesis kit as per the manufacturer’s instruction (Takara). The Syber green-based qPCR was performed using primer sets (Supplementary Table S2) with 50 ng of cDNA from each sample in the QuanStudio 6 Real-time PCR system. The fold change was calculated by the 2^−ΔΔCT^ Method.

## Results

### Synthesis of Lipids

Here, first, we synthesized two intermediate compounds, namely, N,N'-(azanediylbis (ethane-2,1-diyl)distearamide (Intermediate 1A in Supplementary Scheme S1B) and N,N'-((methylazanediyl)bis (ethane-2,1-diyl))distearamide (Intermediate 1B in Supplementary Scheme S1B) by using acid amine coupling *via* imine formation followed by imine reduction with the help of the reducing agent Na(CN)BH_4_. After confirming the intermediate compounds with the help of ESI-MS (Electrospray Ionization–Mass Spectrometry) and 1H-NMR (Supplementary Figures S1−S4) intermediate compounds were reacted with methyl iodide and 2-chloroethanol, resulting in N,N-dimethyl-2-stearamido-N-(2-stearamidoethyl)ethan-1-aminium chloride (lipid DMe18), 2-hydroxy-N-methyl-N,N-bis(2-stearamidoethyl)ethan-1-aminium chloride (lipid MeOH18), and 1-(bis (2-hydroxyethyl) (2-stearamidoethyl)-14-azanyl)-2-stearamidoethan-1-ylium chloride (lipid DOH18). All final compounds were characterized by using ^1^H-NMR, ESI-MS, and HRMS (high-resolution mass spectrometry) (Supplementary Figures S5−S7). The purities of all the final compounds were examined by HPLC (high-performance liquid chromatography), and they were consistently found to be ∼98% pure (Supplementary Figures S8, S12, and S16).

### Physicochemical Characterization of Liposomes

**FIGURE 1:**
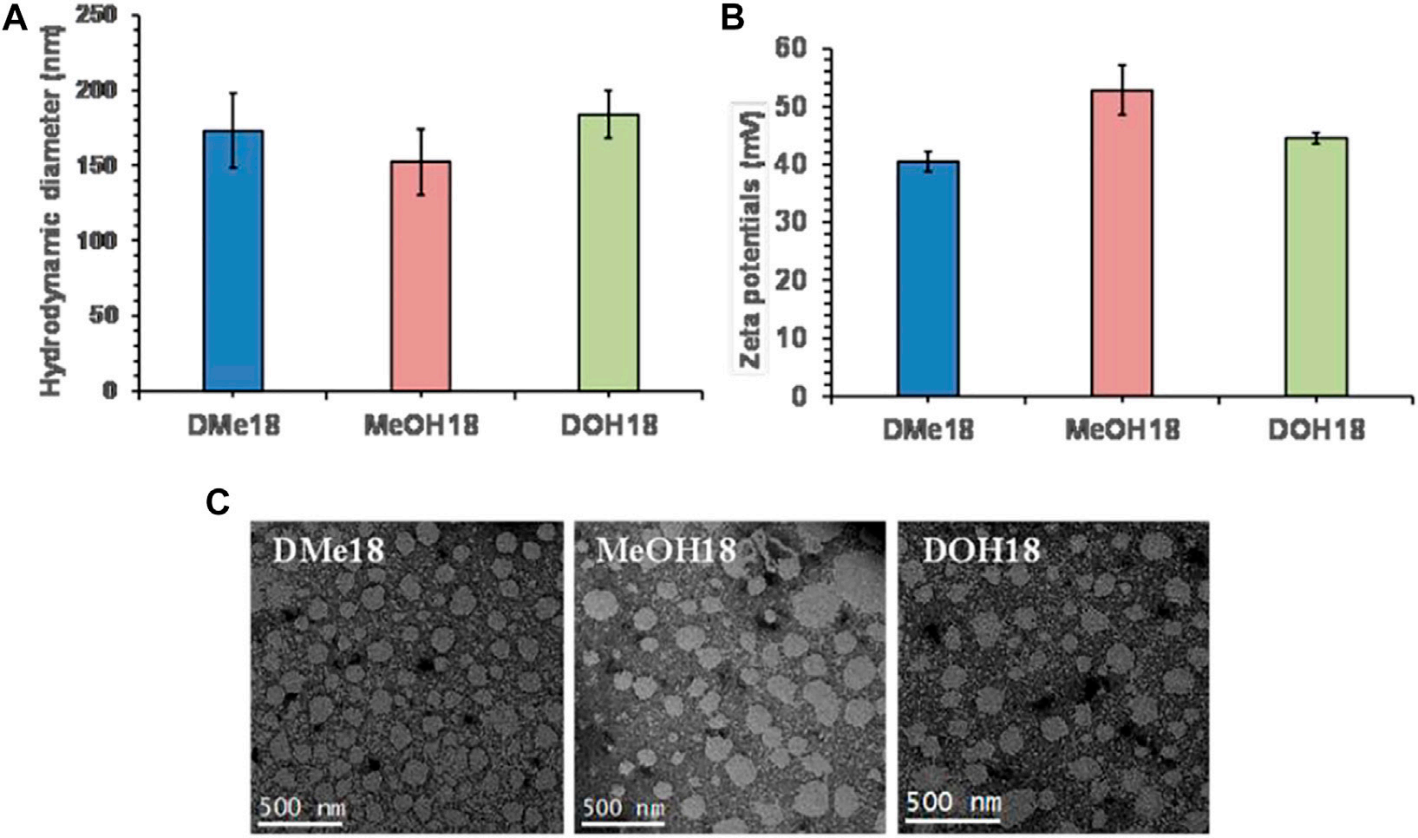
Physicochemical characterization of amide linker-based cationic lipid-associated liposomes. **(A)** Hydrodynamic diameter and **(B)** surface potentials of Lipo-DME, Lipo-MeOH, and Lipo-DOH liposomes in Milli “Q” water and **(C)** transmission electron microscopic analysis of three different liposomal formulations (Scale bar 500 nm).

The liposomes were prepared using amide-based cationic lipids (Supplementary Scheme S1A), DOPE, and cholesterol as co-lipids in the mole ratio of 1:1:1, respectively, *via* the thin-film hydration method. To evaluate the particle size distribution of Lipo-DME, Lipo-MeOH, and Lipo-DOH, we performed the dynamic light scattering analysis of these formulations. We did not observe any significant difference in particle sizes of all three liposomal formulations; they were in the range between 150 and 180 nm hydrodynamic diameter in deionized water (Figure 1A). Furthermore, we observed that the zeta potentials were found to be 40–50 mV (Figure 1B). This indicated that these formulations were cationic in charge and formed strong lipoplexes with the negatively charged nucleic acids. The polydispersity index was found to be 0.26 ± 0.05 for Lipo-DME and 0.25 ± 0.05 for Lipo-MeOH, showing the homogeneity of the formulations. After that we performed the transmission electron microscopic analysis (Figure 1C) of these formulations and observed that all formulations were spherical in shape and uniformly distributed as well as diameters were correlating with the DLS results.

### Characterization of Liposomes

Efficiency of liposomes in forming lipoplexes with pDNA (pLenti-Luciferase-IRES-ZsGreen, BEI #-NR-52516) was evaluated using an agarose gel retardation assay with varying charge ratios of lipid to pDNA from 1:1 to 4:1. All liposomal formulations showed complete lipoplex formation with pDNA in all charge ratios except the Lipo-DME 1:1 ratio, which showed some part of unbound pDNA. Moreover, we have seen that Lipo-DOH lipoplexes form tight complexation with pDNA than other liposomal formulations. Lipo-DOH lipoplexes prevent ethidium bromide intercalation with DNA, and hence signal is less in wells (Supplementary Figure S17A). Furthermore, we evaluated lipoplex stability by the DNase I sensitivity assay. Strong DNA complexation by liposomes prevents DNA from DNase I-mediated degradation. At 2:1 lipid to DNA charge ratio, we observed that all liposomal formulations completely protected DNA from degradation, and the result showed that plasmid was intact and band density was much comparable with naked pDNA control, which was not treated with DNase I (Supplementary Figure S17B). The rationale for varying the hydrophilicity on the hydrophilic head group is to evaluate the optimal ratio of hydrophilic to hydrophobic within the lipid for having efficient pDNA complexation and intracellular localization. We observed that Lipo-DOH showed strong complexation with lenti plasmid system, protected pDNA from enzymatic degradation, and demonstrated superior transfection efficiencies than those of Lipo-DME and Lipo-MeOH and comparable to commercial transfection reagent, Lipofectamine 3000. These findings are in corroboration with previous findings that some of the lipids with the dihydroxy head group were found to be efficient in delivering plasmid DNAs (Venkata Srilakshmi et al., 2002).

### Transfection Efficiency and Cytotoxicity of Liposomes

**FIGURE 2:**
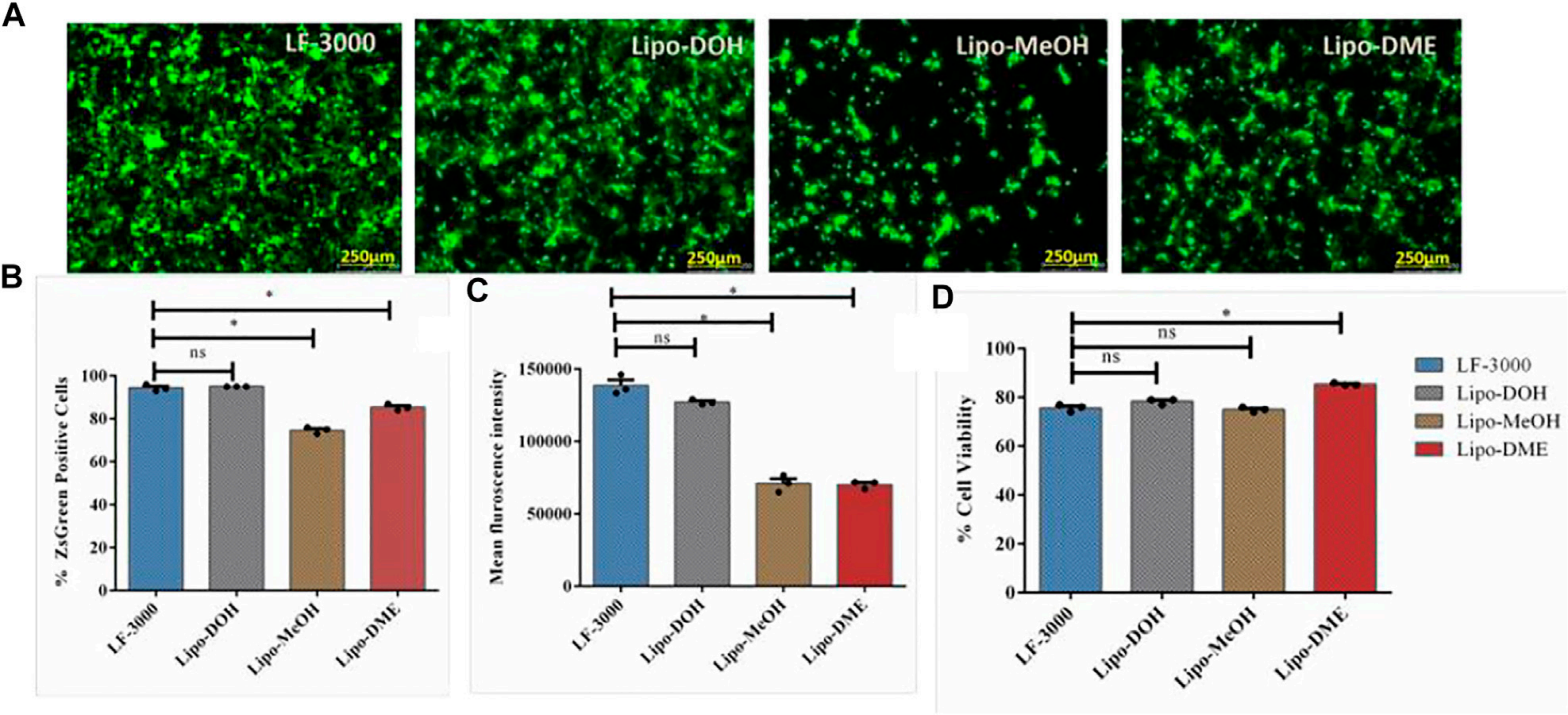
Transfection efficiency of liposomes. HEK-293T cells were transfected by pDNA (Lenti-Luciferase-IRES-ZsGreen) with indicated lipoplexes; the expression of ZsGreen proteins was imaged by using a fluorescent microscope **(A)**, and we quantified percentage of ZsGreen positive cells **(B)** and MFI **(C)** by flow cytometry at 48 h post transfection. At 48 h post transfection, cells were trypsinized and stained with 7-AAD dye and estimated the cell viability by flow cytometry **(D)**.

HEK-293T is the most commonly used cell line for lentiviral production *in vitro*. Hence, we assessed the transfection efficiencies of liposomes in the HEK-293T cell line using the ZsGreen expressing pLenti transfer plasmid (BEI #-NR-52516) with liposomal formation at constant lipid/pDNA charge 2:1 ratio. Lipo-DOH liposome showed maximum transfection efficiency than Lipo-DME and Lipo-MeOH liposomes (Figure 2A,B). Moreover, the Lipo-DOH lipoplex transfected cells showed two-fold higher mean fluorescent intensity of ZsGreen protein than the other two lipoplexes transfected cells at 48 h post-transfection (Figure 2C) and much comparable with the commercial transfection reagent Lipofectamine 3000. Cytotoxic effects of lipoplexes were analyzed using 7-AAD dye staining in HEK-293T cell lines at a 2:1 charge ratio. All lipoplexes transfected cells showed more than 75% of viability as equal to LF-3000 transfected cells (Figure 2D). These results suggested that among our liposomal formulation, Lipo-DOH liposome showed to be an efficient transfection reagent which equals to the LF-3000 transfection reagent in HEK-293T cells. Hence, we selected the Lipo-DOH liposome lentiviral-based SARS-CoV-2 spike-pseudovirus production in HEK-293T cells.

### Human ACE2 Receptor Overexpressing HEK-293T Cell Line Development

**FIGURE 3:**
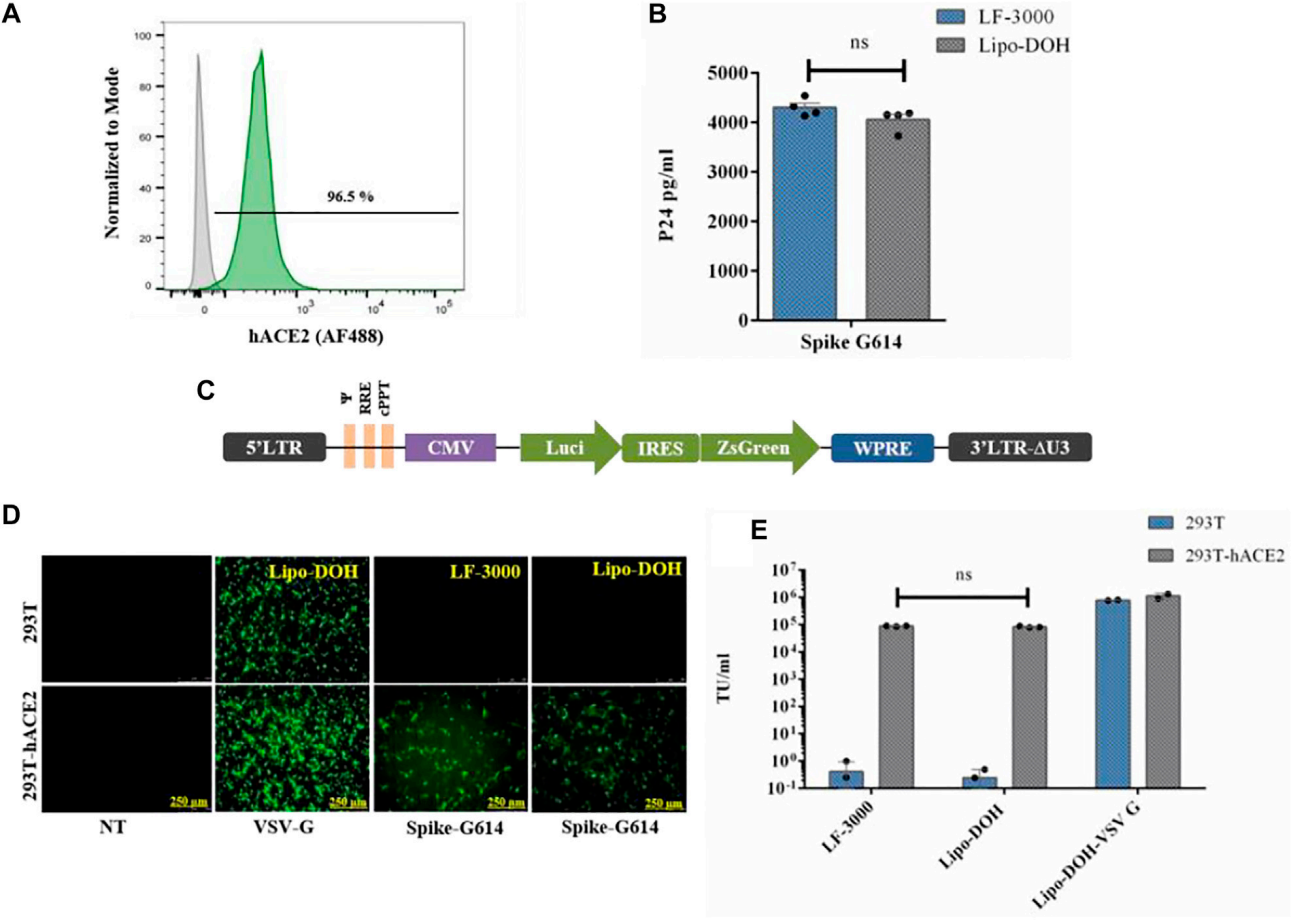
Comparative study of spike G614 pseudovirus production and functional efficiency by LF-3000 and Lipo-DOH liposome delivery system. Expression of hACE2 protein in 293T-hACE2 stable cell line was confirmed by immunostaining followed by flow cytometry **(A)**. Pseudovirus produced in HEK-293T cells by LF-3000 and Lipo-DOH and the quantified level of pseudovirus by p24 ELISA **(B)**. The vector map of Lenti transfer plasmid which is used in this study **(C)**. The functional titer of pseudovirus was performed into 293T and 293T-hACE2 cells at different dilution of viral supernatants. At 72-h post transduction, pseudoviruses infectivity was imaged using a fluorescent microscope (image shows infectivity of VSV-G pseudovirus at 1/10^th^ dilution and spike-G614 at 1/2nd dilution) **(D)**, and transduction unit was calculated from ZsGreen positive cells that were quantified by flow cytometry. **(E)** ns, non-significant between groups. Data mean ± SEM.

The SARS-CoV-2 virus infects human cells through ACE2 receptors and cell surface proteases (Furin and TMPRSS2) (Hoffmann et al., 2020a). Hence, we developed a 293T-hACE2 cell line constitutively expressing hACE2 protein by lentiviral vector from HEK-293T (293T) cells. We used an in-house made lentiviral vector expressing hACE2 and puromycin under EF1α promoter for the development of 293T-hACE2 cell line. A stable cell line was made and selected by puromycin for four passages. At five passages, above 95% of cells had high expression of hACE2 protein when compared to 293T cells (Figure 3A).

### Pseudovirus Production Using Liposomes

For identifying the best lentiviral plasmids system for pseudovirus production, we chose two commonly used lentiviral plasmids systems one from Addgene (second generation) and another from BEI for production of spike-G614 and VSV-G pseudovirus by Lipo-DOH liposome in HEK-293T cells. For pseudovirus production, we used the lentiviral transfer plasmid (pLenti- Luciferase-IRES-ZsGreen, BEI #-NR-52516) as it has both ZsGreen and luciferase reporter genes. This makes it easy to monitor the pseudovirus functional titer by flow cytometry as well as pseudovirus infectivity by the luciferase assay (Figure 3C). The yield and functional titer (TU) of pseudovirus were estimated by p24 ELISA and flow cytometry, respectively. The results showed that the level of p24 protein was significantly higher in both spike-G614 and VSV-G pseudovirus produced by BEI than in the Addgene lentiviral production system, and the functional titer of these pseudoviruses also increased in the BEI system (Supplementary Figures S18A,B).

The spike G614 pseudovirus production efficacy of Lipo-DOH and LF-3000 were evaluated in HEK-293T cells. The Lipo-DOH liposome produced an equal level of spike G614 pseudovirus as LF-3000 liposome (Figure 3B). As we expected, the produced spike G614 pseudovirus infection was high in hACE2 overexpressing 293T-hACE2 cells than in 293T cells, but broad-spectrum VSV-G pseudovirus infected efficiently both 293T and 293T-hACE2 cells. This is confirmed by both fluorescent imaging as well as TU tire flow cytometry (Figure 3D,E). Moreover, the functional titer of produced spike G614 pseudovirus in 293T-hACE2 cells showed equal efficiency as LF-3000 in 293T-hACE2 cells (Figure 3E).

Invading of SARS-CoV-2 virus into cells requires cleavage of spike protein by cell surface proteases (hTmprss2) during infection in humans (Hoffmann et al., 2020b). Hence, we also studied whether overexpression of hTmprss2 enhances the infectivity of spike G614 pseudovirus in 293T-hACE2 cells by analyzing luciferase expression. The high expression of hACE2 and hTmprss2 mRNA was confirmed by qPCR in 293T-hACE2-hTmprss2 cells (Supplementary Figure S19A). We observed that high expression of hTmprss2 could not increase the significant enhancement of spike G614 pseudovirus infectivity (Supplementary Figure S19B). This observed result revealed that endogenous expressing hTmprss2 proteins in 293T cells might be sufficient to processing of spike G614 protein for infection of spike G614 pseudovirus. Furthermore, we found there was no significant difference in infectivity of spike G614 pseudovirus produced by both Lipo-DOH and LF-3000 systems in 293T-hACE2 and 293T-hACE2+hTmprss2 cells (Supplementary Figure S19B).

### Evaluation of Spike G614 Pseudovirus for Neutralization Assay

**FIGURE 4:**
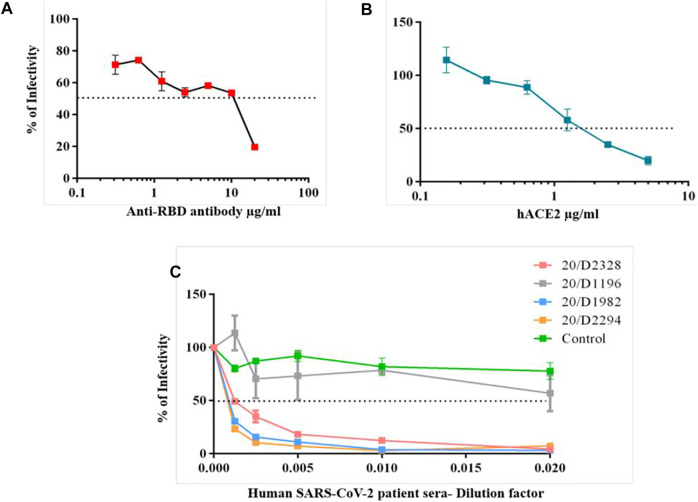
Spike G614 pseudovirus neutralization assay. The spike G614 pseudovirus was neutralized with the anti-RBD antibody **(A)**, soluble hACE2 protein **(B)**, and human SARS-CoV-2 patient sera **(C)** at different dilutions. After 1 h of incubation, spike G614 pseudovirus was infected into 293T-hACE2 cells for 72 h. Later, the percentage of infectivity was calculated by the luciferase assay.

First, we performed a spike G614 pseudovirus neutralization assay with commercially available anti-RBD antibody (P06DHuRb) and soluble hACE2 protein. Both antibody and hACE2 protein effectively neutralized the spike G614 pseudovirus infectivity. The antibody at above ∼10 μg/ml concentration showed inhibitory concentrations of 50% (IC50), whereas hACE2 protein efficiently neutralize even at above ∼1 μg/ml (Figure 4A,B). These data revealed that spike G614 mutant protein has more affinity to hACE2 protein, thus the hACE2 protein neutralizes more spike G614 pseudovirus at a lower concentration than the RBD antibody. This could be due to G614 mutation in the spike protein of pseudovirus which was well proven to have stronger interactions. Finally, we validated pseudovirus with IgG against spike positive sera from individuals recovered from COVID-19.

Before the evaluation of the spike G614 pseudovirus neutralization assay using human SARS-CoV-2 patient sera, we performed a trimeric spike-hACE2 blocking assay to find the levels of spike antibodies blocking the binding of spike-hACE2 in human patient sera samples at different dilution. We found that all the SARS-CoV-2 patient sera samples have different levels of spike blocking antibodies when compared to control sera (Supplementary Figure S19). Among that 20/D2328, 20/D1985, and 20/D2294 patient sera have a high level of spike blocking antibodies than other sera samples (Supplementary Figure S19). Based on this study, we selected 20/D2328, 20/D1985, 20/D2294, and 20/D1196 (have less spike blocking antibodies) sera samples and used them for the spike G614 pseudovirus neutralization assay.

All three selected sera samples (20/D2328, 20/D1985, and 20/D2294) effectively neutralized spike G614 pseudovirus and showed above IC80 value at 1/200 serum dilution. Moreover, we observed that the samples (20/D1985 and 20/D2294) showed around IC70 even at 1/800 serum dilution (which is the highest dilution used in this study), whereas the 20/D2328 sample showed IC50 at 1/800 serum dilution (Figure 4C). As we expected, 20/D1196 did not show IC50 even at the lowest dilution (1/50) (Figure 4C).

## Discussion

Choice of the liposomal system is critical for lentivirus production. Liposomal gene delivery has the advantage of having good transfection efficiency with lower cytotoxicity than cationic polymers. However, delivering plasmids over 10 kb is always a challenging task with the liposomal system (Meisel and Gokel, 2016). More importantly, all the plasmids used for lentivirus production have more than 5 kb, which results in low titer with liposomes (Jiang et al., 2015). To overcome this challenge, we designed amide linker-based cationic lipids, varying their head group from dimethyl (DME18) to methyl, ethylhydroxyl (MeOH18), and diethylhydroxyl (DOH18) keeping lipophilic stearic acid twin chains as constant. Typically, cationic lipid consists of a hydrophilic head group, hydrophobic tail, and linker functionality. In our prior findings, we demonstrated that amide linker lipids showed superior transfection efficiencies than ester linker based lipids and provided protection to DNA from enzymatic degradation (Srujan et al., 2011). The rationale for varying the hydrophilicity on the hydrophilic head group is to evaluate the optimal ratio of hydrophilic to hydrophobic within the lipid for having efficient pDNA complexation and intracellular localization. We observed that Lipo-DOH showed strong complexation with the lenti plasmid system, protected pDNA from enzymatic degradation, and demonstrated superior transfection efficiencies than Lipo-DME and Lipo-MeOH and comparable to the commercial transfection reagent Lipofectamine 3000. These findings are in corroboration with previous findings that some of the lipids with the dihydroxy head group found to be efficient in delivering plasmid DNAs.

To evaluate the transfection efficiencies, ZsGreen as a reporter lentiviral transfer plasmid system, which is 9 kb in size, was used. As the other lentiviral plasmids, including the packaging plasmid and envelope plasmids, are in a similar size range, we anticipated a similar efficiency of transfection with the efficient liposomal reagent Lipo-DOH for making lentiviruses. In lines with the transfection results with reporter plasmid ZsGreen, we observed similar efficiencies of pseudovirus production of Lipo-DOH compared to Lipofectamine 3000.

Prior reports demonstrated that pseudovirus of SARS-CoV-2 expressing surface glycoprotein could be used as an alternative to the live virus for neutralization assays (Knudson et al., 2021; Schmitz et al., 2021). In line with the prior findings, we made pseudovirus with the most common virus used in gene transfer studies, lentivirus. For optimizing the lentivirus production, we compared the Addgene lentiviral plasmid system, which consists of a three plasmid system, with the BEI lentiviral plasmid system, which consists of five plasmids. In both the plasmid systems, envelope and transfer plasmids are the same but packaging plasmids are different. Addgene has psPAX2 with a size of ∼11 kb as the packaging plasmid, whereas BEI has three different plasmids**,** namely, HDM-Hgpm2 (NR-52517), pRC-CMV-Rev1b (NR-52519), and HDM-tat1b (NR-52518) with sizes ∼4–8 kb. The size of the plasmid directly correlates to the expression in the transfections. Plasmids below 10 kb have better nuclear delivery than plasmids above 10 kb as their nuclear entry is compromised that in turn affect their transfection properties. Taking cues from the literature, we hypothesized that due to the lower size of packaging plasmids, BEI could have produced high titer lentivirus than the Addgene system. Furthermore, we used the BEI lentiviral production system for spike-G614 pseudovirus production. The spike G614 mutant protein increases virion density and infectivity of SARS-CoV2 virus through high-affinity binding toward the hACE2 receptor than wild-type spike D614 protein (Baric, 2020; Korber et al., 2020; Zhang et al., 2020; Daniloski et al., 2021). Thus, we selected spike G614 mutant for the production of pseudovirus in this study. When we compared the titer values of pseudovirus produced with commercial Lipofectamine 3000 and Lipo-DOH, we observed similar p24 values, and infectivity in hACE2 expressing HEK-293T cell line revealed that our liposomal reagent was as efficient as the commercial one in delivering larger plasmids of the lentivirus system and that in turn resulted in producing high titer pseudovirus. The produced pseudovirus was validated using the RBD antibody and soluble hACE2 protein. We observed in the neutralization assay that pseudovirus showed a stronger interaction to hACE2 than the RBD antibody. This could be due to G614 mutation in the spike protein of pseudovirus which was well proven to have stronger interactions. Finally, we validated pseudovirus with IgG against spike positive sera from individuals recovered from COVID19.

**FIGURE 5:**
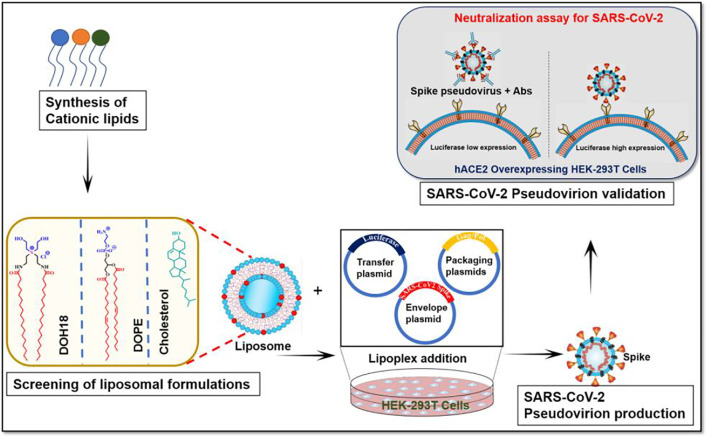
Schematic presentation of the protocol that includes the steps involved in producing SARS-CoV-2 pseudovirion.

In summary, for optimizing conditions for making SARS-CoV-2 pseudovirus in lentivirus backbone, first, we designed and synthesized cationic lipids for conducting a structure–activity investigation on varying head groups to identify the efficient transfection reagent. With the efficient transfection reagent, we evaluated two different lentiviral plasmid systems from Addgene and BEI and concluded that the lentiviral system from BEI showed a higher titer of lentivirus. Finally, the pseudovirus was functionally validated its infectivity in the hACE2 cell line and neutralization efficiency in presence of hACE2 and RDB antibody ELISA (Figures 5). The comprehensive protocol can be further explored for high-throughput screening of neutralizing antibodies patient population who are vulnerable to SARS-CoV-2 reinfection of multiple variants.

## Data Availability

The original contributions presented in the study are included in the article/Supplementary Materials, further inquiries can be directed to the corresponding author.
