# Supplementary material for: Optimization of SARS-CoV-2 Pseudovirion Production in Lentivirus Backbone With a Novel Liposomal System
Source: Front Pharmacol. 2022 Mar 25;13:840727. doi: 10.3389/fphar.2022.840727 (PMC8990231; doi:10.3389/fphar.2022.840727)
Supplement: Supplementary file 1 [file DataSheet1.docx]

Optimization of SARS-CoV-2 pseudovirion production in lentivirus backbone with a novel liposomal system

*Gokulnath Mahalingam^1^, Hari Krishnareddy Rachamalla^2^, Porkizhi Arjunan^1^, Yogapriya Periyasami^1^, Salma M^1^, Rajkumar Banerjee^2^, Saravanabhavan Thangavel. Kumarasamypet M Mohankumar^1^, Mahesh Moorthy^3^, Shaji R Velayudhan^1^, Alok Srivastava^1^, Srujan Marepally^†*^*

1. Centre for Stem Cell Research (CSCR) (a unit of inStem, Bengaluru), CMC Campus, Vellore,

632002, TN, India

2. CSIR-Indian Institute of Chemical Technology, Tarnaka, Hyderabad, 506007, India

3. Department of Clinical Virology, Christian Medical College, Vellore, 632004, TN, India

**Supplementary information**

**Synthetic procedures of amide linker based cationic lipids**

**
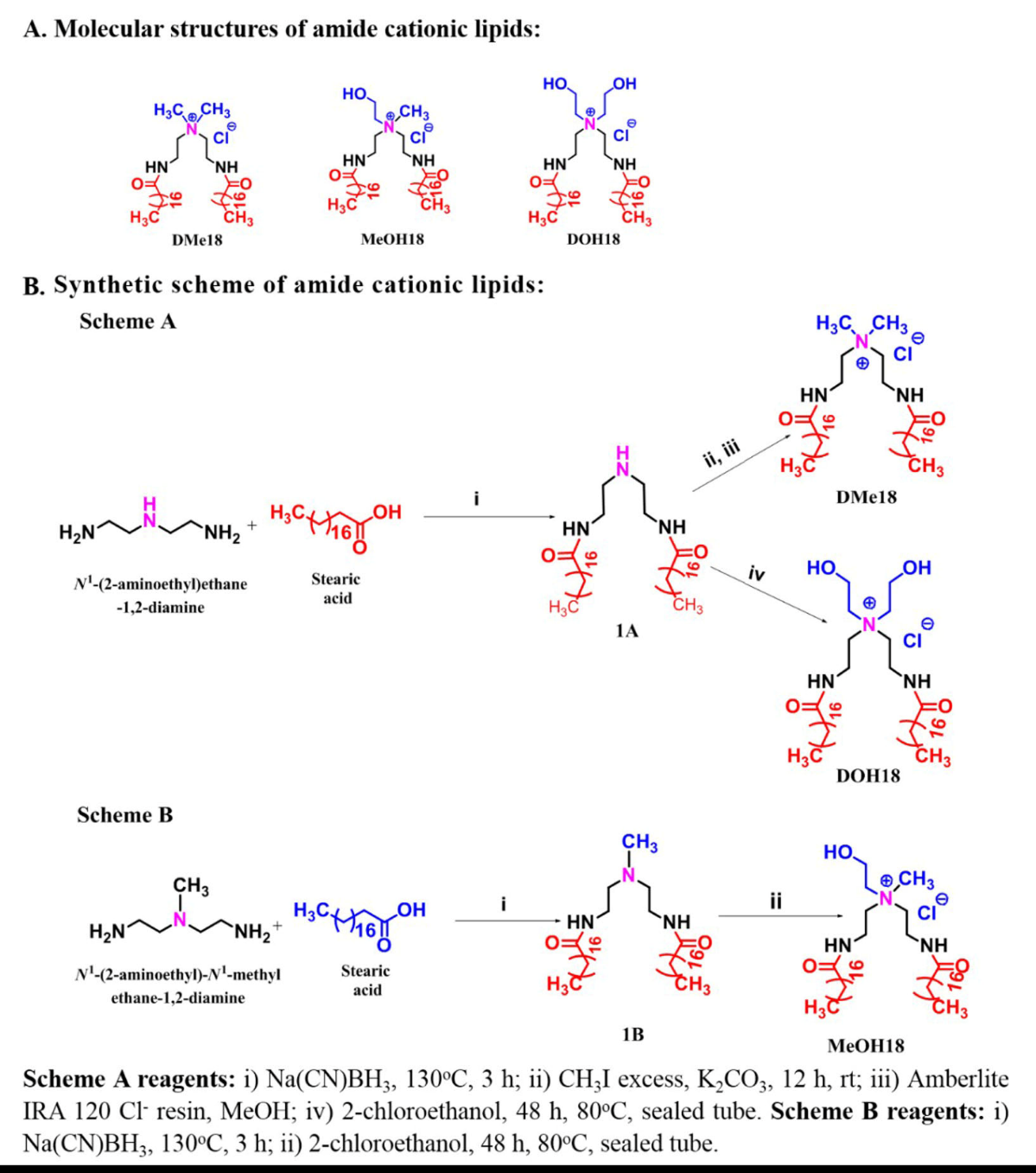
**

**Scheme S1:** Molecular structures (A) and synthetic scheme (B) of three amide-linker based cationic lipids changing hydrophilic head group with constant hydrophobic stearic acid twin chain (DMe18, MeOH18 and DOH18).

**Synthesis of N, N-dimethyl-2-stearamido-N-(2-stearamidoethyl)ethan-1-aminium chloride (lipid DMe18, Scheme A):**

**Step i: N, N'-(azanediylbis(ethane-2,1-diyl))distearamide (Intermediate 1A in Scheme A):**

Stearic Acid (1 g, 3.51 mmol) was stirred in 100 ml round bottom flask at 130^o^C till it liquefies. To this N^1^-(2-aminoethyl)ethane-1,2-diamine (0.14g, 1.35 mmol) was added under stirring, after 5 min catalytic amount of sodium cyanoborohydride (0.01 g, 0.19 mmol) was added. This reaction mixture was further stirred for 3 h at 130^o^C. The crude compound was purified by column chromatography using 60-120 mesh silica gel in chloroform/methanol 99:1(v/v), afforded pure 1A intermediate. (0.85 g, 72 % yield, Rf = 0.7 in 5% methanol in chloroform).

**^1^H NMR of (CDCl_3_, 400 MHz):** δ/ppm = 0.88 [t, 6H, C**H**_3_-(CH _2_)_16_-]; 1.27 [m, 56H, CH_3_-(C**H**_2_)_14_-CH_2_-CH_2_-]; 1.65 [m, 4H, CH_3_-(CH _2_)_14_-C**H**_2_-CH_2_-]; 2.20[t, 4H, CH_3_-(CH _2_)_14_-CH_2_-C**H**_2_-]; 2.50 2.78[t,4H, -CO-NH-CH_2_-C**H**_2_-NH-C**H**_2_-CH_2_-NH-CO-]; 3.36[t, 4H, -CO-NH-C**H**_2_-CH_2_-NH-CH_2_-C**H**_2_-NH-CO-]; 6.05[t, 2H, -CO-N**H**-CH_2_-CH_2_-NH-CH_2_-CH_2_-N**H**-CO-],

**ESI-MS m/z:** calcd636.11 (for C_40_H_81_N_3_O_2_), found 637 [M]^+^

**Step ii, iii: Synthesis of N, N-dimethyl-2-stearamido-N-(2-stearamidoethyl)ethan-1-aminium chloride (DMe18):**

Intermediate 1A (0.2 g, 0.31 mmol) prepared in step *I* (**Scheme A)** was dissolved in 1mL of DCM in a 25mLround-bottom ﬂask, and1mL of methyl iodide was added to the solution. The reaction mixture was stirred at room temperature for 12 h and concentrated. The crude compound was puriﬁed by column chromatography using 60-120 mesh silica gel in chloroform/methanol 95:5 (v/v). The pure compoundupon chloride ionexchange onAmberlite-IRA120 resin (using methanol as eluent) afforded pure DMe18 lipid as a white solid (0.17 g, 79 % yield, Rf = 0.5 in 5% methanol in chloroform).

**^1^H NMR of (CDCl_3_, 400 MHz):** δ/ppm = 0.87 [t, 6H, C**H**_3_-(CH _2_)_16_-]; 1.24 [m, 56H, CH_3_-(C**H**_2_)_14_-CH_2_-CH_2_-]; 1.60 [m, 4H, CH_3_-(CH _2_)_14_-C**H**_2_-CH_2_-];2.27 [t, 4H, CH_3_-(CH _2_)_14_-CH_2_-C**H**_2_-]; 3.29 [S, 6H, -CH_2_-CH_2_-N^^(C**H**_3_)_2_-CH_2_-CH_2_-]; 3.79 [t, 8H, -CO-C**H**_2_-C**H**_2_-N^^(CH_3_)_2_-C**H**_2_-C**H**_2_-CO-]; 7.53[t, 2H, -N**H**-CO-CH_2_-CH_2_-N^^(CH_3_)_2_-CH_2_-CH_2_-CO-N**H**-],

**ESI-MS m/z:** calcd665.17 (for C_42_H_86_N_3_O_2_^+^), found 665[M]^+^

**ESI-HRMS:** 664.6711(calculated mass forC42 H86 O2 N3 = 664.6715)

**HPLC- Purity:** 98.22%

**Step iv: Synthesis of 1-(bis (2-hydroxyethyl)(2-stearamidoethyl)-l4-azanyl)-2-stearamidoethan-1-ylium chloride (lipid DOH18, Scheme 1.A):**

Intermediate 1A (0.2 g, 0.38 mmol, prepared above in step i in scheme A) in sealed tube, excess 2-chloro ethanol (0.18 g, 2.29 mmol) was added in sealed tube as a solvent. The reaction mixture was stirred at 80^o^C for 48 h. Excess 2-chloro ethanol was removed on rotary evaporator. The residue upon column chromatographic puriﬁcation (using 60-120 mesh size silica gel and 5:95 methanol:chloroform, v/v, as eluent) afforded pure DOH18 as a white solid (0.14 g, 58 % yield, Rf = 0.2, in 5 % methanol in chloroform).

**^1^H NMR of (CD_3_OD, 400 MHz):** δ/ppm =0.84 [t, 6H, C**H**_3_-(CH _2_)_12_-]; 1.24 [m, 40H, CH_3_-(C**H**_2_)_10_-CH_2_-CH_2_-]; 1.54 [m, 4H, CH_3_-(CH _2_)_10_-C**H**_2_-CH_2_-]; 2.16 [t, 4H, CH_3_-(CH _2_)_10_-CH_2_-C**H**_2_-]; 3.25 [t, 4H, -CO-NH-CH_2_-CH_2_-N^^(C**H**_2_-CH_2_-OH)_2_-CH_2_-CH_2_-NH-CO-]; 3.55 [t, 4H, -CO-NH-CH_2_-C**H**_2_-N^^(CH_2_-CH_2_-OH)_2_-CH_2_-CH_2_-NH-CO-]; 3.61 [t, 4H, -CO-NH-C**H**_2_-CH_2_-N^^(CH_2_-CH_2_-OH)_2_-CH_2_-CH_2_-NH-CO-]; 3.99 [m, 4H, -CO-NH-CH_2_-CH_2_-N^^ (CH_2_-C**H**_2_-OH)_2_-CH_2_-CH_2_-NH-CO-]; 4.53 [S, 2H, -CO-NH-CH_2_-CH_2_-N^^(CH_2_-CH_2_-O**H**)_2_-CH_2_-CH_2_-NH-CO-];

**ESI-MS m/z:** calcd725.22 (for C_44_H_89_ClN_3_O_4_^+^), found 725[M]^+^

**ESI-HRMS:** 724.6914 (calculated mass forC_44_H_89_ClN_3_O_4_^+^=724.6926)

**HPLC- Purity:** 98.05%

**Synthesis of 2-hydroxy-N-methyl-N, N-bis(2-stearamidoethyl)ethan-1-aminium chloride (lipid MeOH18, Scheme B):**

**Step i: N, N'-((methylazanediyl)bis(ethane-2,1-diyl))distearamide (Intermediate 1B in Scheme B):**

Stearic Acid (1 g, 3.51 mmol) was stirred in 100 ml round bottom flask at 130^o^C till it liquifies. To this liquid N^1^-(2-aminoethyl)ethane-1,2-diamine (0.16 ml, 1.15 mmol) was added, and catalytic amount of sodium cyanoborohydride (0.01 g, 0.17 mmol) was added after 5 min and the reaction mixture was further stirred for another 3 h at 130^o^C. The crude compound was purified by column chromatography using 60-120 mesh silica gel in chloroform/methanol 99.5:0.5(v/v). (0.75 g, 80% yield, Rf = 0.7 in 5% methanol in chloroform)

**^1^H NMR of (CDCl_3_, 400 MHz):** δ/ppm = 0.87 [t, 6H, C**H**_3_-(CH _2_)_16_-]; 1.25 [m, 40H, CH_3_-(C**H**_2_)_14_-CH_2_-CH_2_-]; 1.61 [m, 4H, CH_3_-(CH _2_)_14_-C**H**_2_-CH_2_-]; 2.19 [t, 4H, CH_3_-(CH _2_)_14_-CH_2_-C**H**_2_-]; 2.24 [s, 3H, , -CO-NH-CH_2_-CH_2_-NC**H**_3_-CH_2_-CH_2_-NH-CO-]; 2.50 [t,4H, -CO-NH-CH_2_-C**H**_2_-NH-C**H**_2_-CH_2_-NH-CO-]; 3.34 [t, 4H, -CO-NH-C**H**_2_-CH_2_-NH-CH_2_-C**H**_2_-NH-CO-]; 6.05[t, 2H, -CO-N**H**-CH_2_-CH_2_-NH-CH_2_-CH_2_-N**H**-CO-],

**ESI-MS m/z:** calcd650.13 (for C_41_H_83_N_3_O_2_), found 651[M]^+^

**Step ii: Synthesis of 2-hydroxy-N-methyl-N, N-bis(2-stearamidoethyl)ethan-1-aminium chloride (lipid MeOH18, Scheme 1.B):**

Intermediate 1B (0.2 g, 0.37 mmol, prepared above in step iii) in sealed tube, excess 2-chloro ethanol (0.17 g, 2.11 mmol) was added in sealed tube as a solvent. The reaction mixture was stirred at 80^o^C for 48 h. Excess2-chloro ethanol was removed on rotary evaporator. The residue upon column chromatographic puriﬁcation (using 60-120 mesh size silica gel and 5:95 methanol:chloroform, v/v, as eluent) afforded pure MeOH14 as a white solid (0.16 g, 78% yield, Rf = 0.4 in 5% methanol in chloroform).

**^1^H NMR of (CD_3_OD, 400 MHz):** δ/ppm = 0.88 [t, 6H, C**H**_3_-(CH _2_)_12_-]; 1.23 [m, 40H, CH_3_-(C**H**_2_)_10_-CH_2_-CH_2_-]; 1.54 [m, 4H, CH_3_-(CH _2_)_10_-C**H**_2_-CH_2_-]; 2.18 [t, 4H, CH_3_-(CH _2_)_10_-CH_2_-C**H**_2_-]; 3.24[S,3H, -CO-NH-CH_2_-CH_2_-N^^(C**H**_3_)(CH_2_CH_2_OH)-CH_2_CH_2_-NH-CO-]; 3.34 [t, 2H, -CO-NH-CH_2_-CH_2_-N^^(CH_3_)(C**H**_2_CH_2_OH)-CH_2_CH_2_-NH-CO-]; 3.48 [t, 4H, -CO-NH-CH_2_-C**H**_2_-N^^(CH_3_)(CH_2_CH_2_OH)-C**H**_2_CH_2_-NH-CO-]; 3.59 [t, 4H, -CO-NH-C**H**_2_-CH_2_-N^^(CH_3_)(CH_2_CH_2_OH)-CH_2_C**H**_2_-NH-CO-]; 3.81[[t, 2H, -CO-NH-C**H**_2_-CH_2_-N^^(CH_3_)(CH_2_CH_2_OH)-CH_2_C**H**_2_-NH-CO-]; 3.96 [S,1H, , -CO-NH-CH_2_-CH_2_-N^^(CH_3_)(CH_2_CH_2_O**H**)-CH_2_CH_2_-NH-CO-];

**ESI-MS m/z:** calcd695.20 (for C_43_H_88_ClN_3_O_3_^+^), found 695[M]^+^

**ESI-HRMS:** 694.6809 (calculated mass for

**HPLC- Purity:** 98.29%

**Spectral characterization data for amide cationic lipids**

**(^1^H NMR, ESI-MS, HRMS and HPLC)**


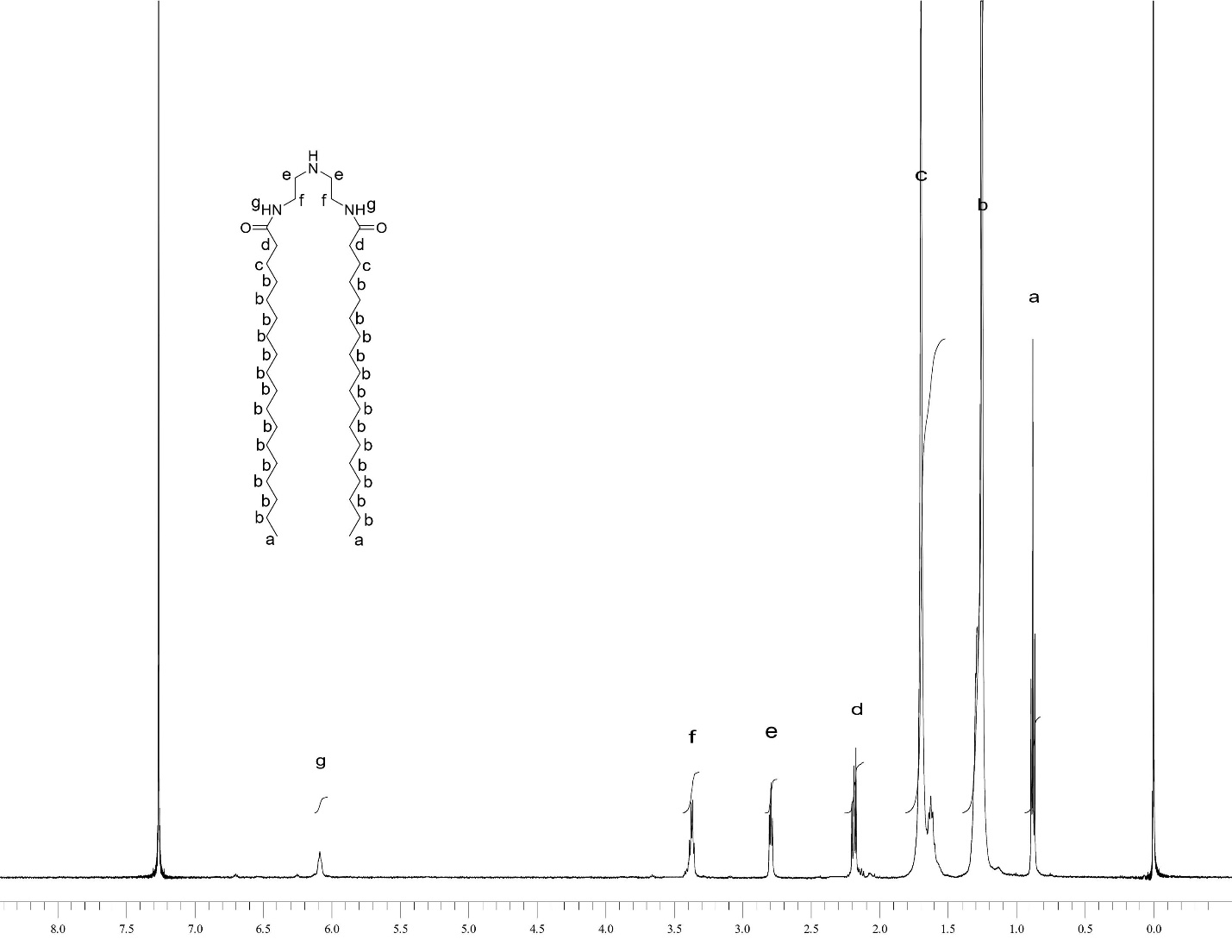


**Figure S1:** ^1^H NMR data of intermediate 1A


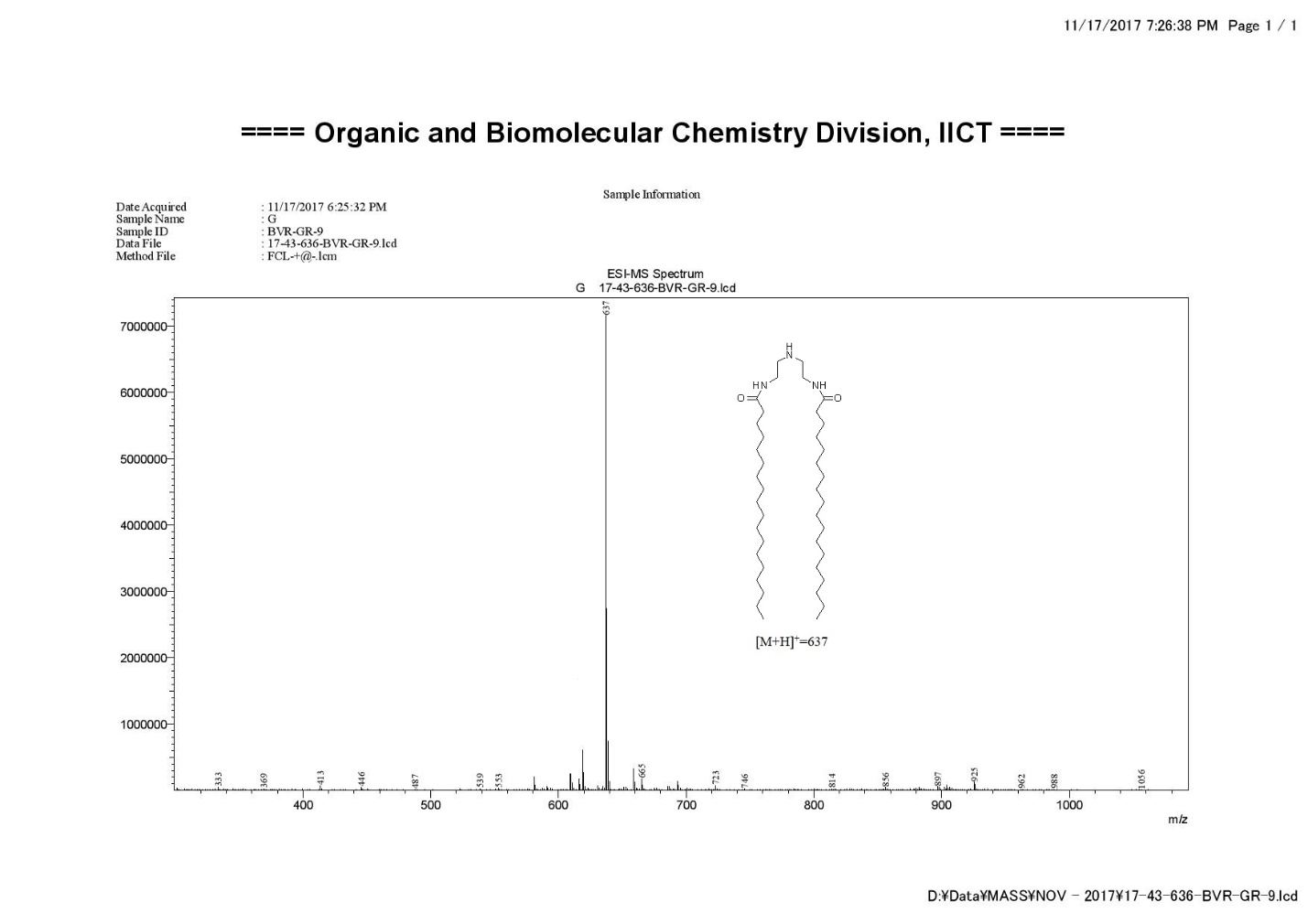


**Figure S2:** ESI-MS data of intermediate 1A


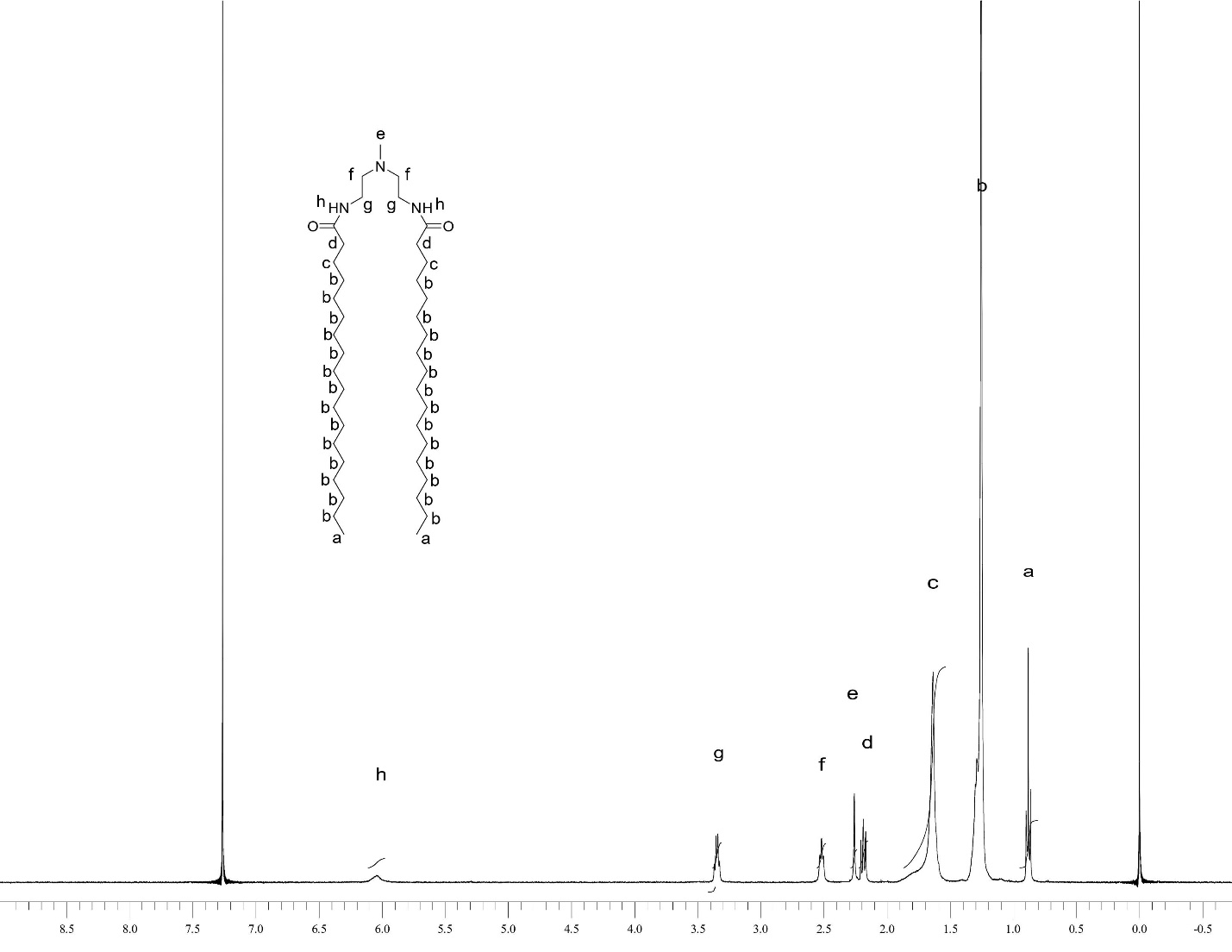


**Figure S3:** 1H NMR data of intermediate 1B


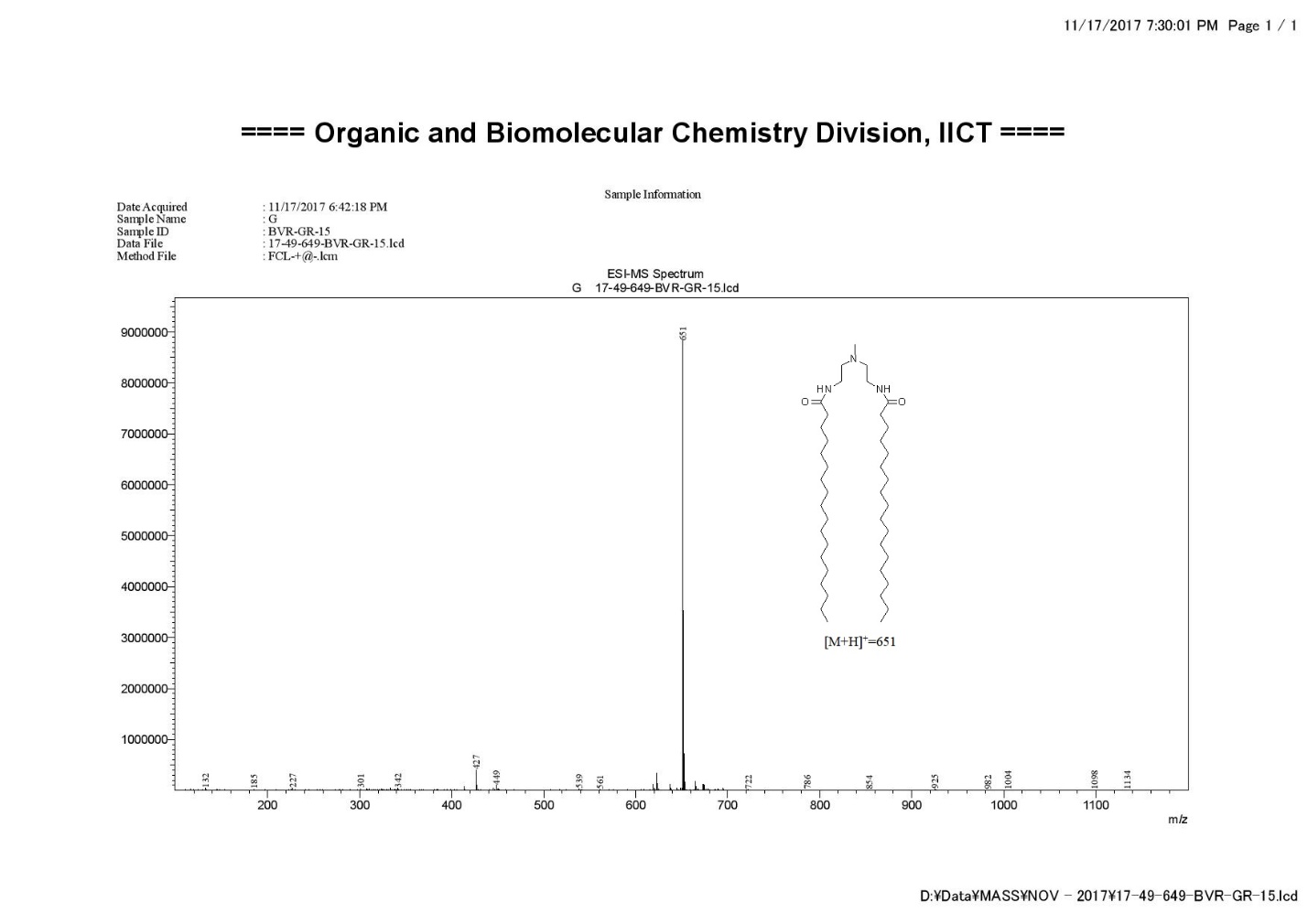


**Figure S4:** ESI-MS data of intermediate 1B

**
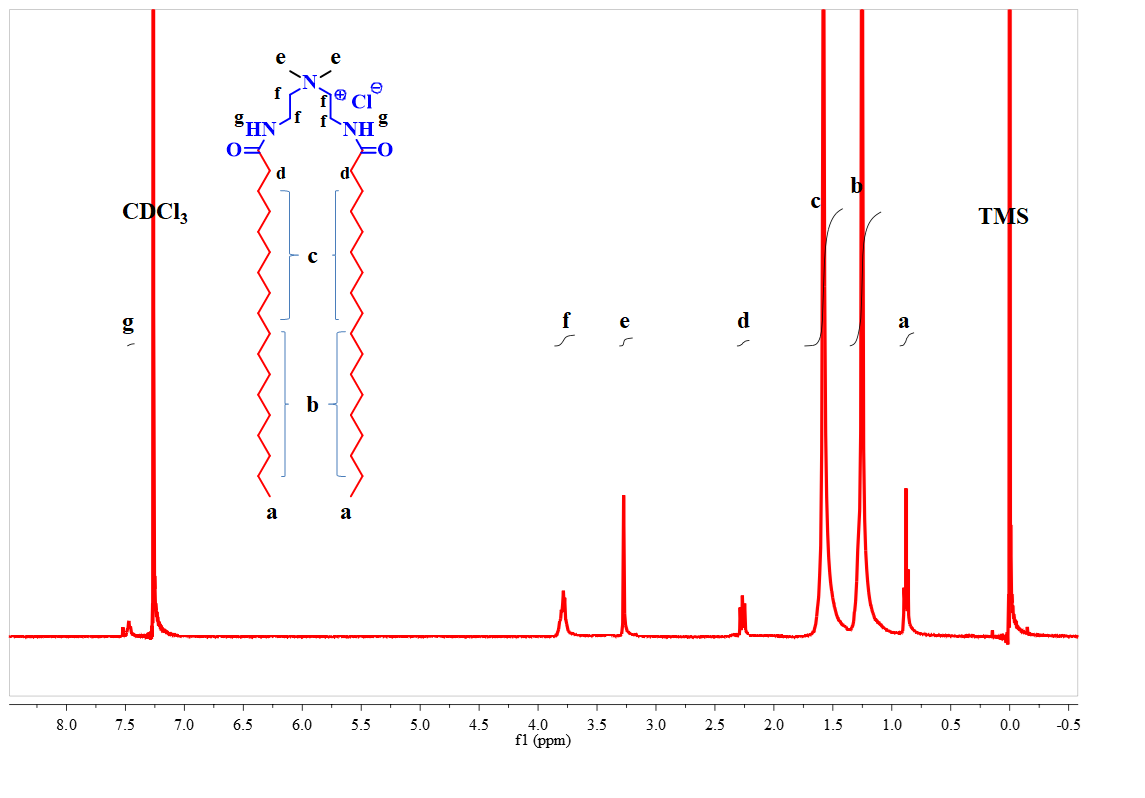
**

**Figure S5:** ^1^H NMR data of DMe18

**
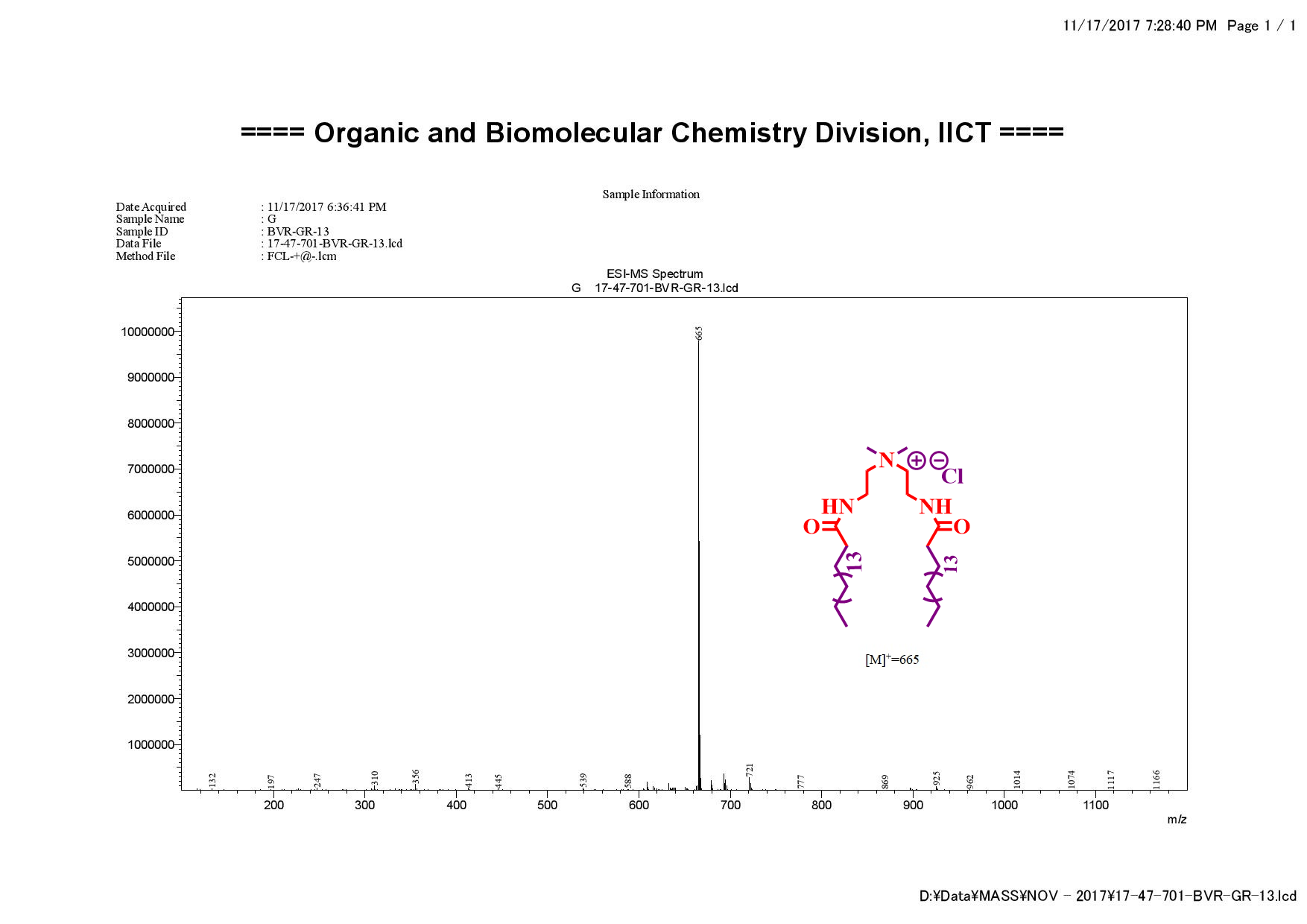
**

**Figure S6:** ESI-MS data of DMe18

**
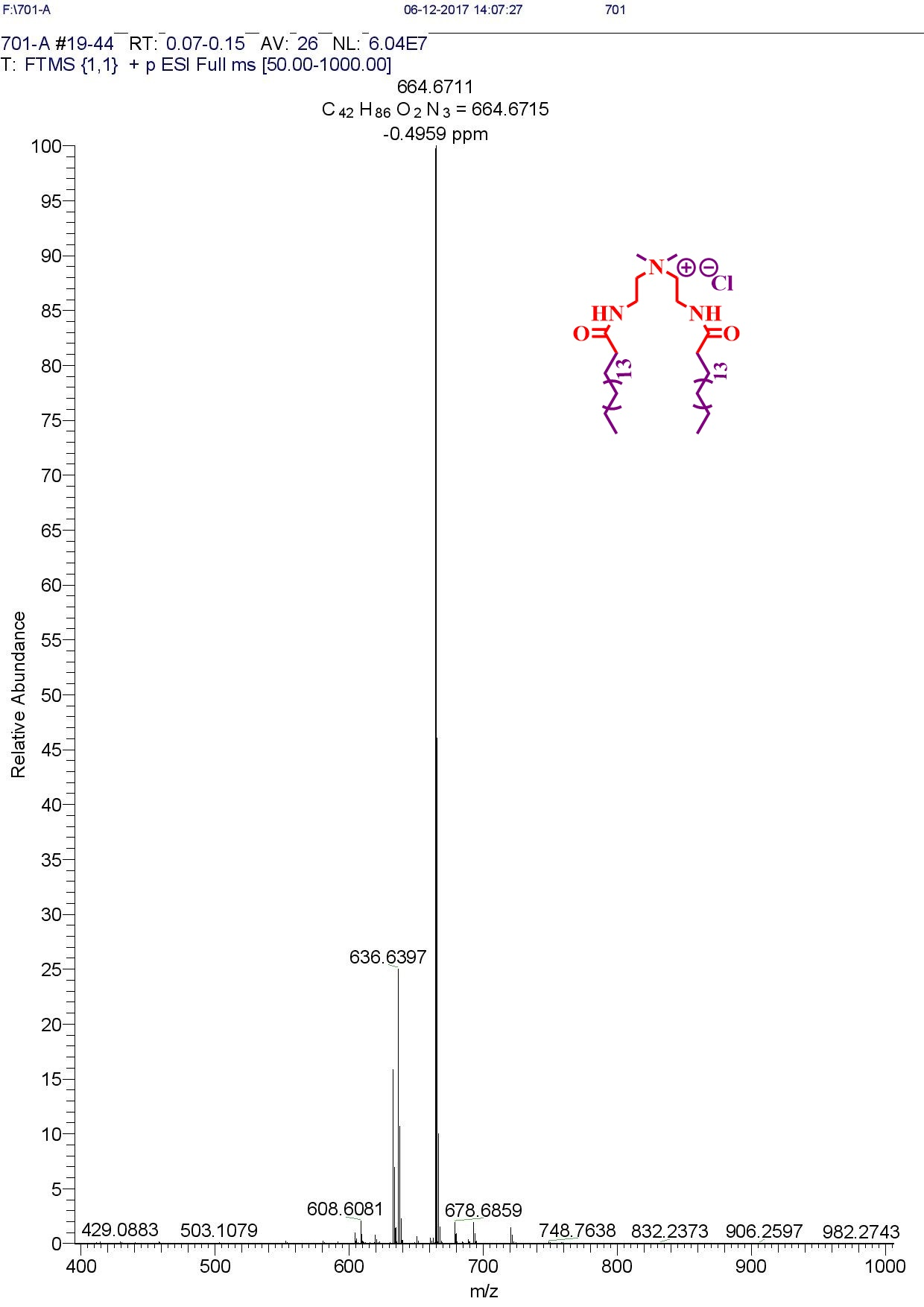
**

**Figure S7:** ESI-HRMS data of DMe18

**
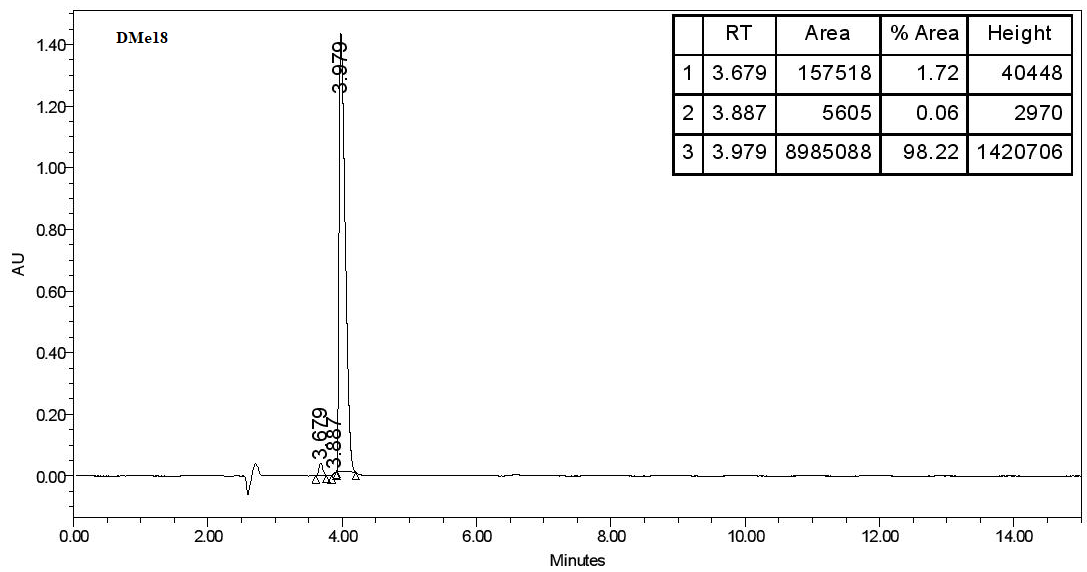
**

**Figure S8:** HPLC data of DMe18

**
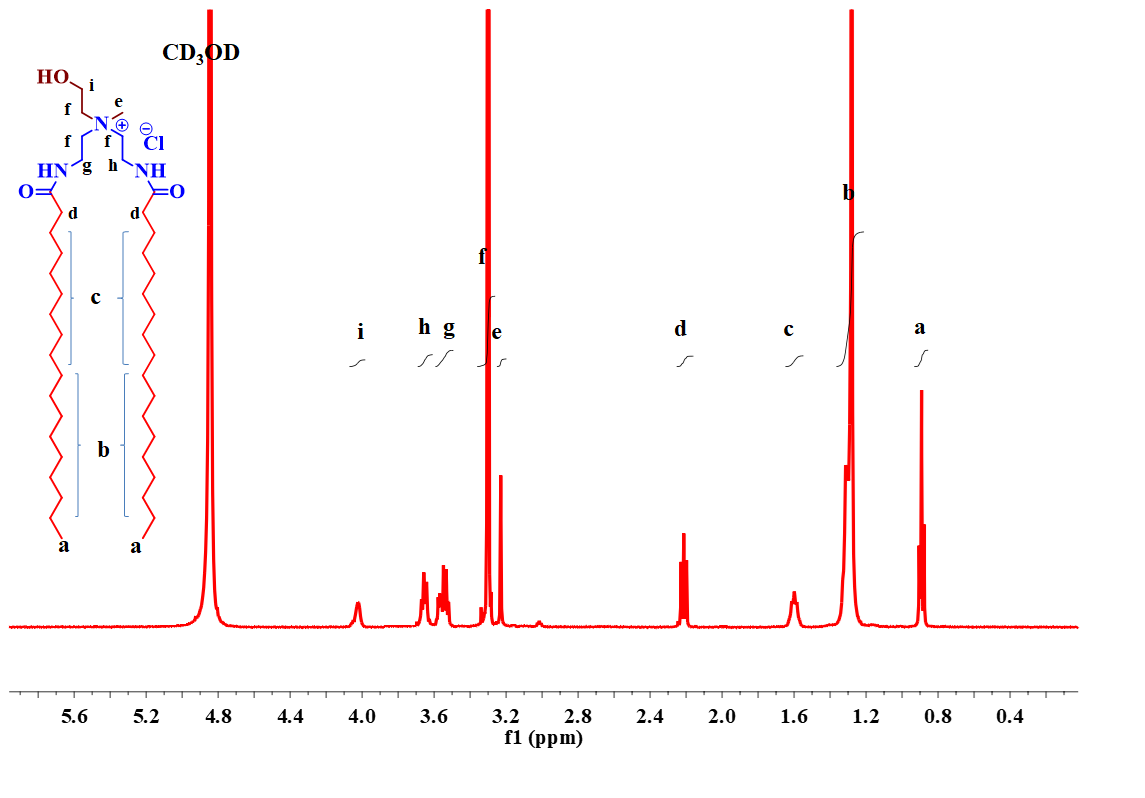
**

**Figure S9:** ^1^H NMR data of MeOH18

**
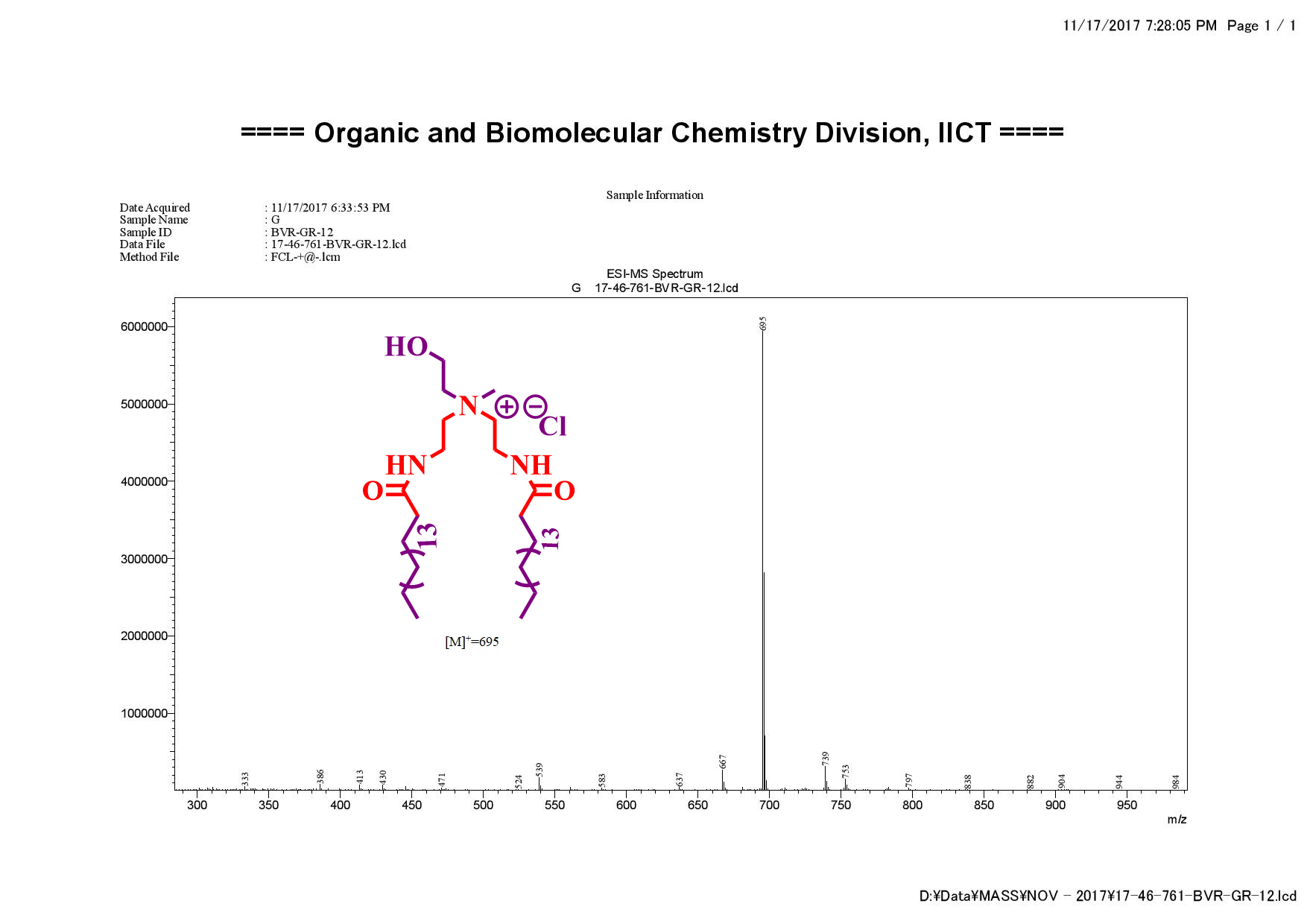
**

**Figure S10:** ESI-MS data of MeOH18

**
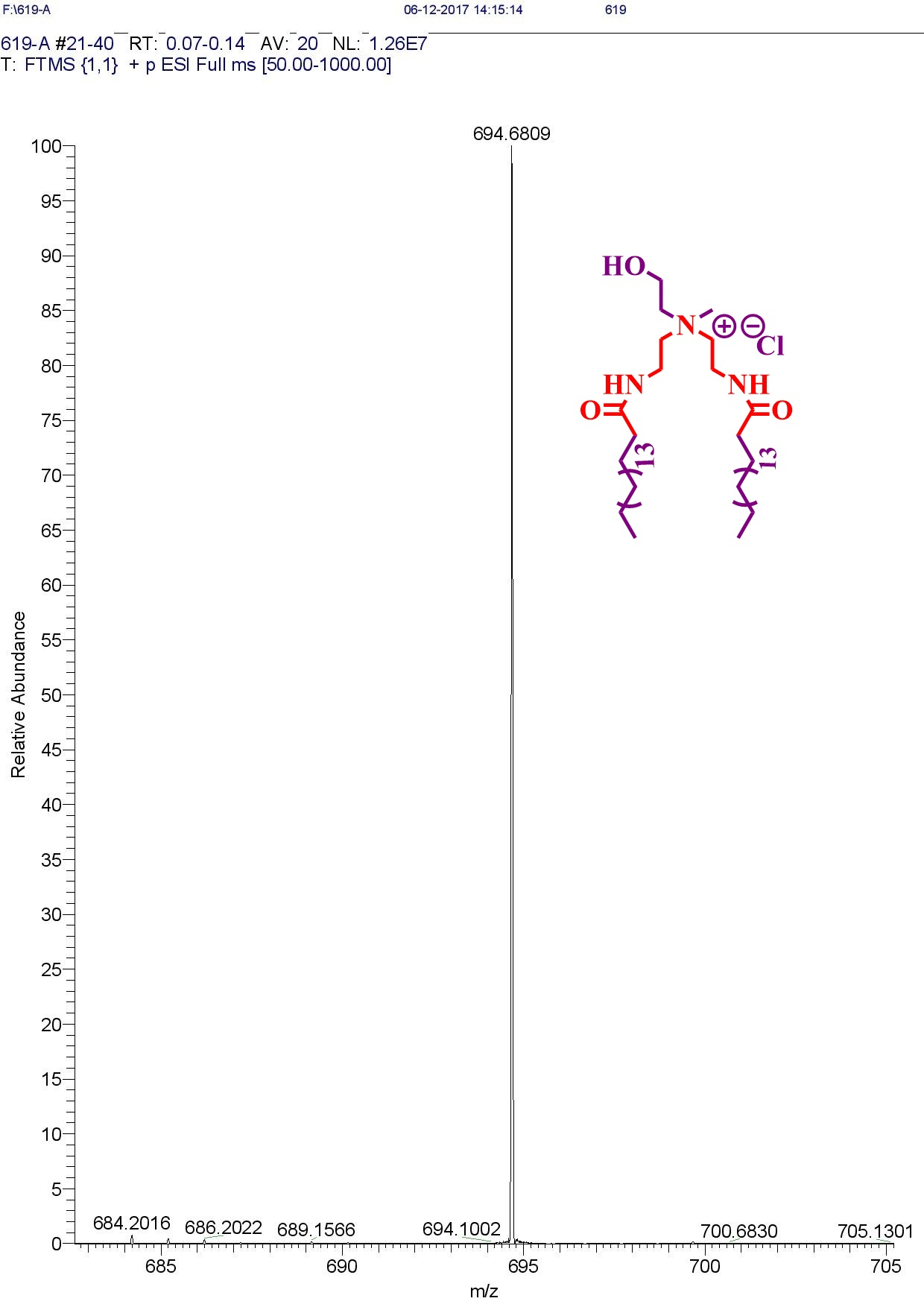
**

**Figure S11:** ESI-HRMS data of MeOH18

**
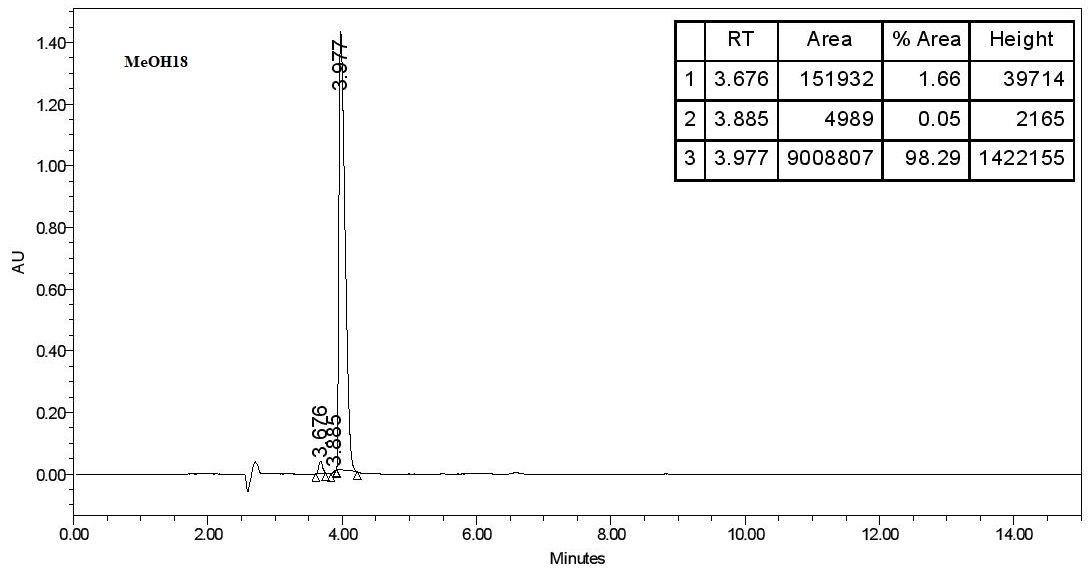
**

**Figure S12:** HPLC data of MeOH18

**
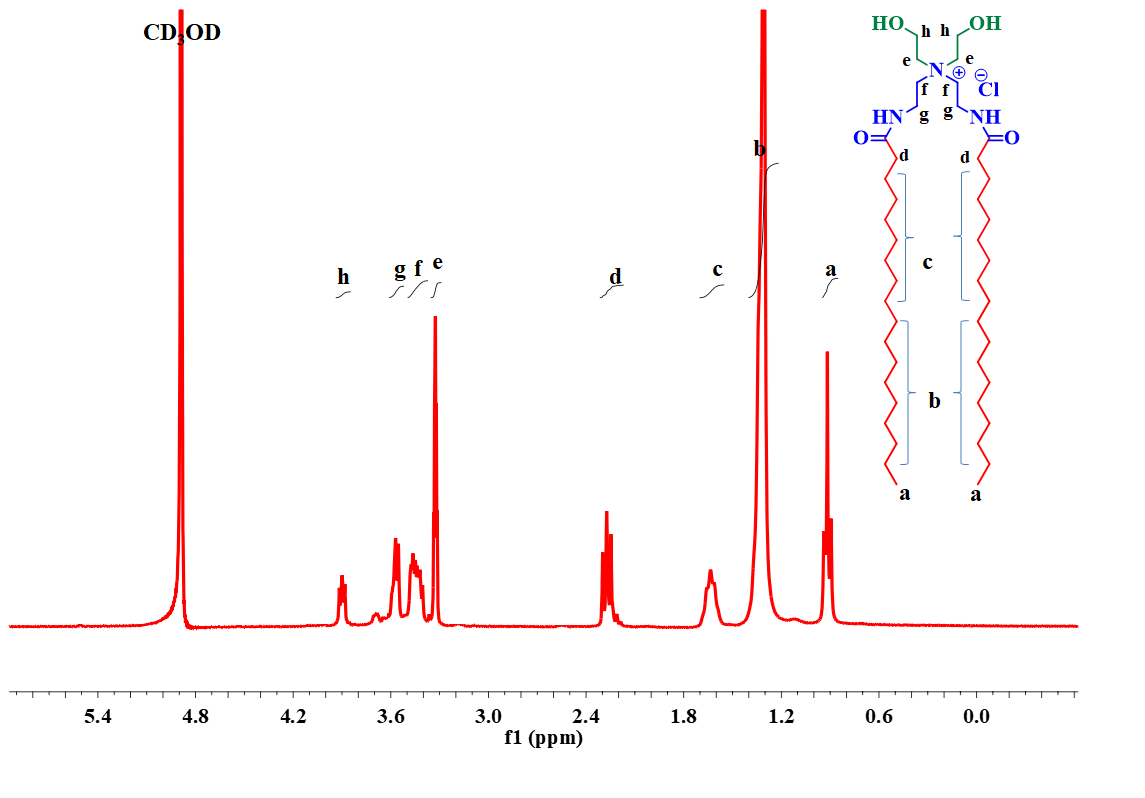
**

**Figure S13:** ^1^H NMR data of DOH18

**
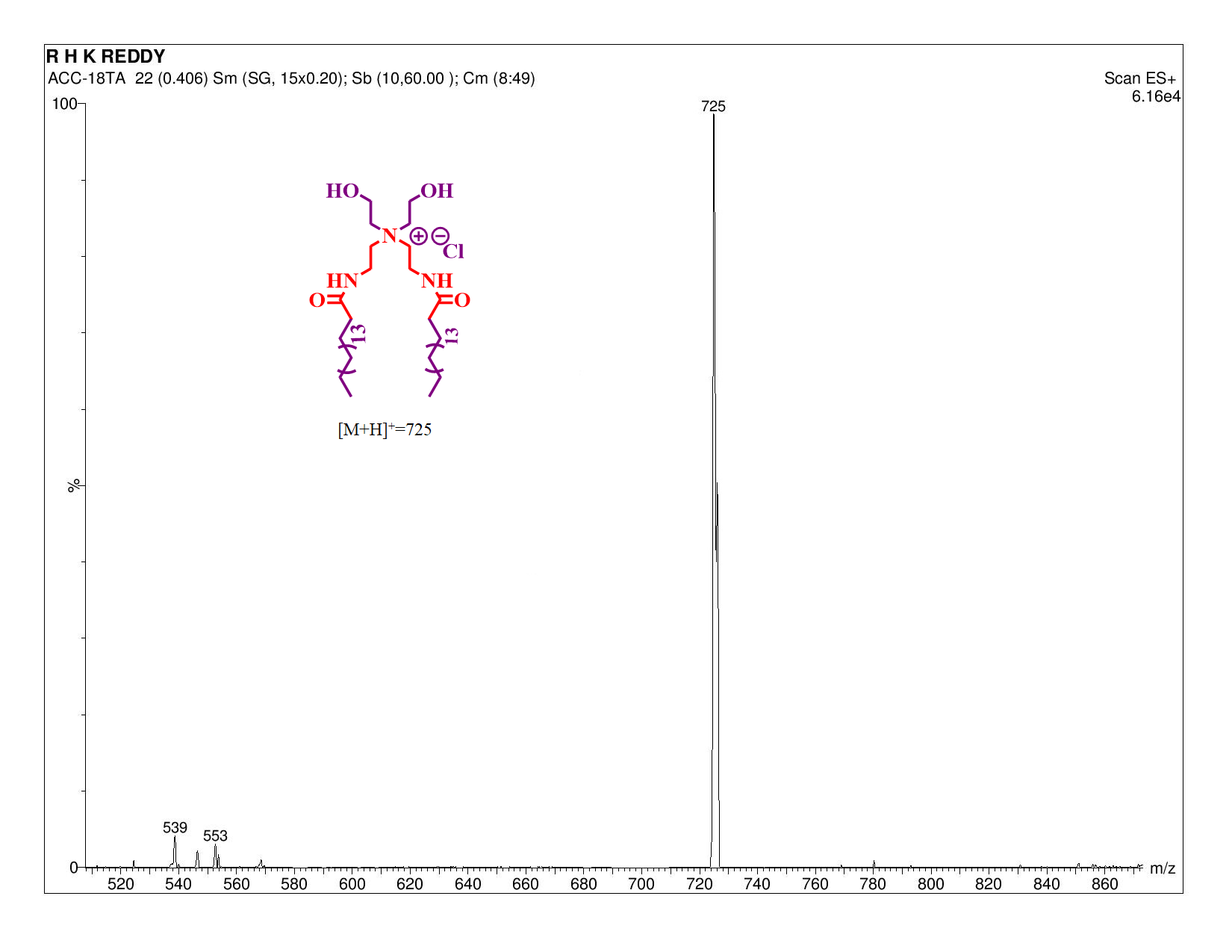
**

**Figure S14:** ESI-MS data of DOH18

**
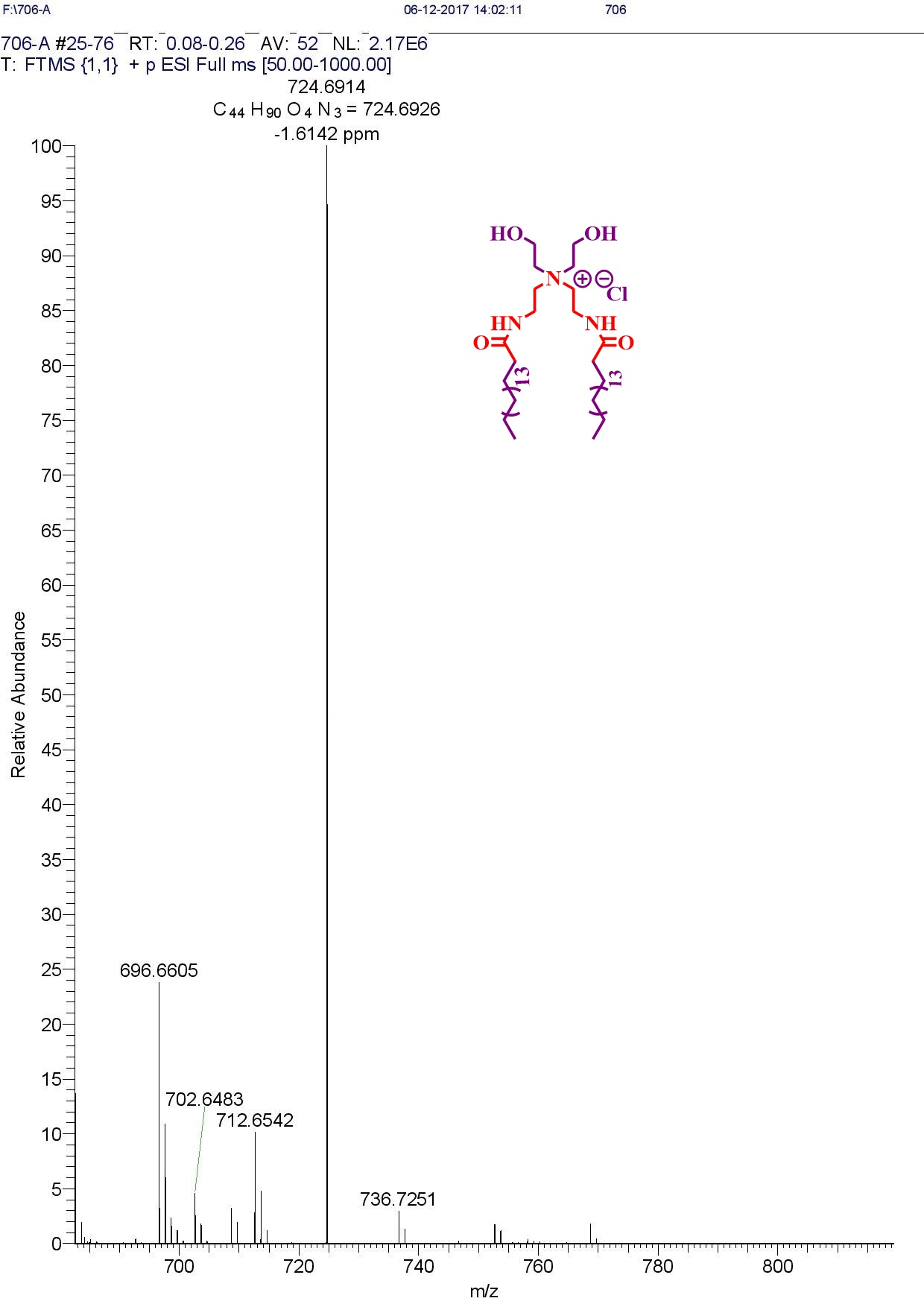
**

**Figure S15:** ESI-HRMS data of DOH18

**
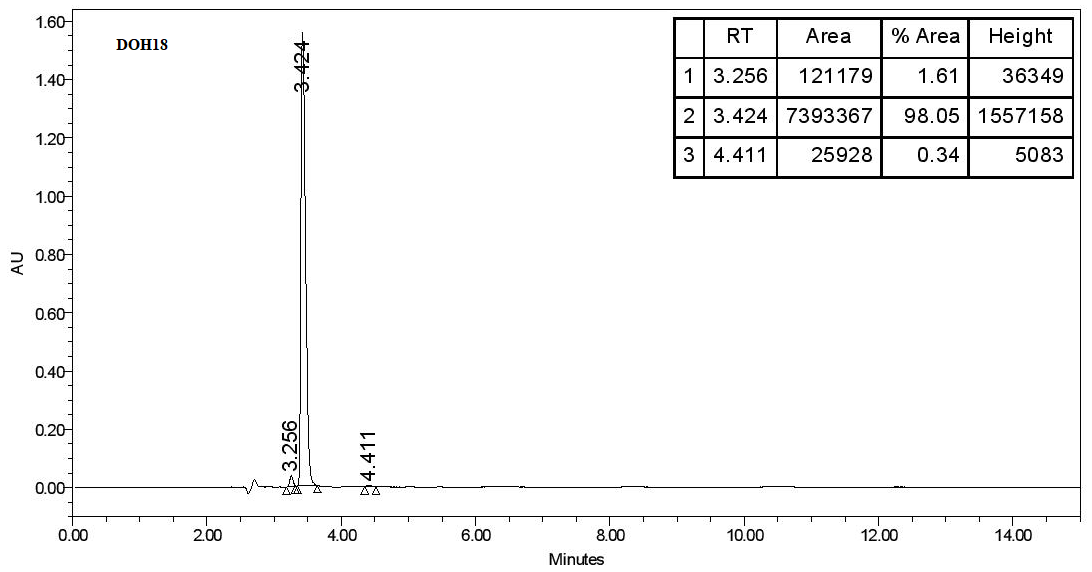
**

**Figure S16:** HPLC data of DOH18

**HPLC Conditions:**

System: Varian Prostar series

Column: Lichrospher® 100, RP-18e (5 μm)

Mobile Phase: Methanol

Flow Rate: 1.0 mL/min

Typical Column Pressure: 72 Bars

Detection: UV at 210 nm


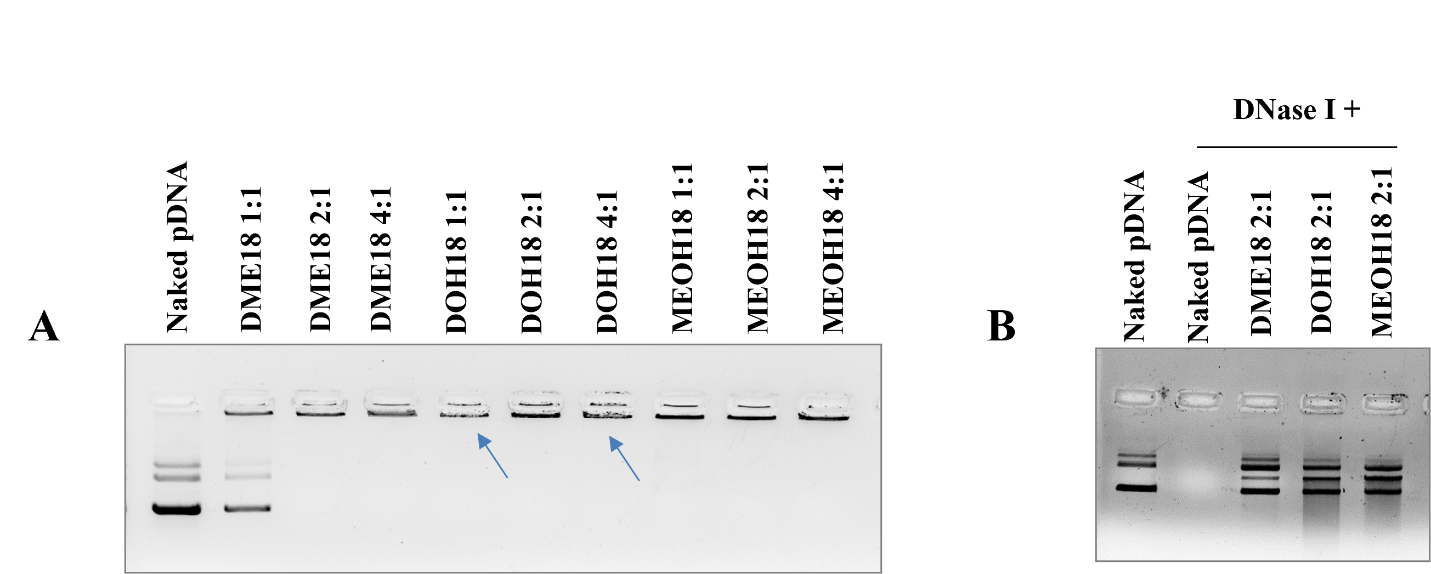


**Figure S17**. Analysis of trapping efficiency and protection of pDNA (Lenti-transfer plasmid) by Liposomes. Gel Retardation assay with different charge ratio of pDNA and Liposomes (A) and DNase I protection assay with Lipoplexes (B).


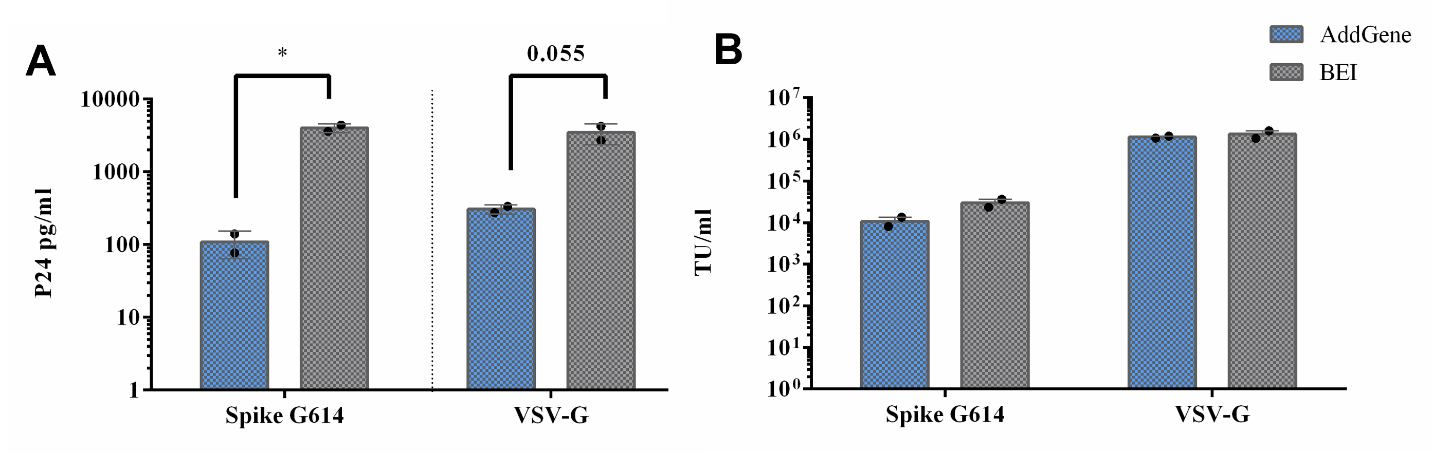


**Figure S18.** Production efficiency of pseudovirus by Addgene and BEI Lentiviral plasmids system with Lipo-DOH liposome. The spike-G614 and VSV-G pseudovirus were produced with Addgene and BEI Lentiviral plasmids system in HEK-293T cells. The level of these pseudoviruses were estimated by quantifying the p24 proteins in harvested viral supernatants by ELISA (A) and functional titre of pseudovirus was performed in 293T-hACE2 cells at 72 h post transduction by flow cytometry (B). * Indicated a significant higher in BEI lentiviral production system when compared to Addgene lentiviral production system. Data mean + SEM


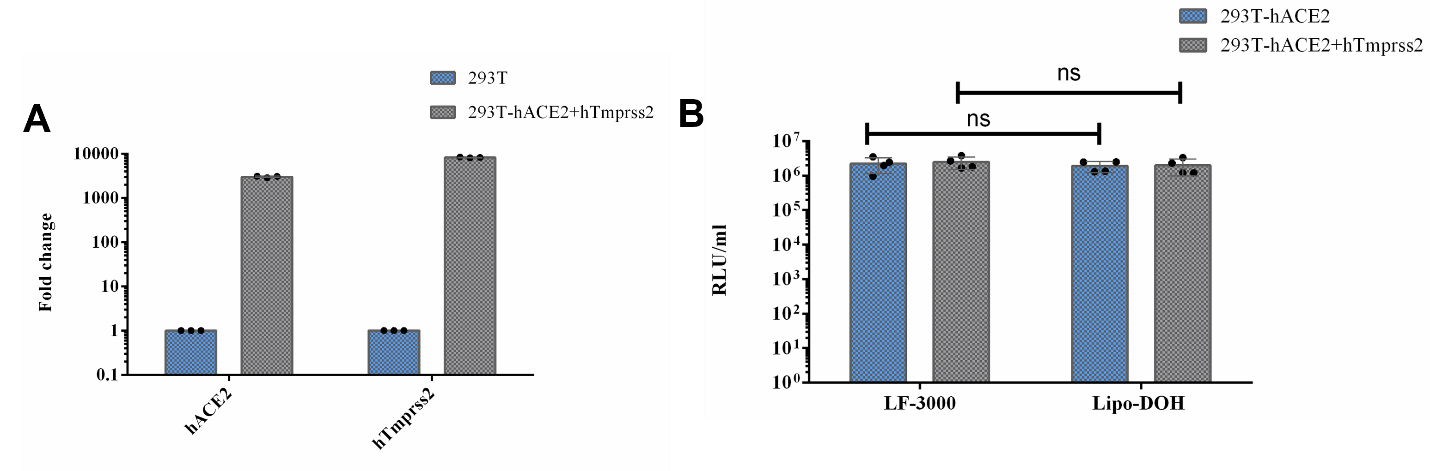


**Figure S19.** Quantification of Spike G614 pseudovirus infectivity by luciferase assay. The 293T-hACE2 cells transiently transfected with pTmprss2 plasmid, 12 hours before Spike G614 pseudovirus transduction. High expression of hACE2 and hTmprss2 mRNA were quantified by qPCR and fold change was calculated by normalized with these mRNAs expression in 293T cells (A). At 72 h post spike G614 pseudovirus infectivity, the luciferase assay was performed and quantified RLU/ml (B). ns, non-significant between groups. Data mean + SEM.


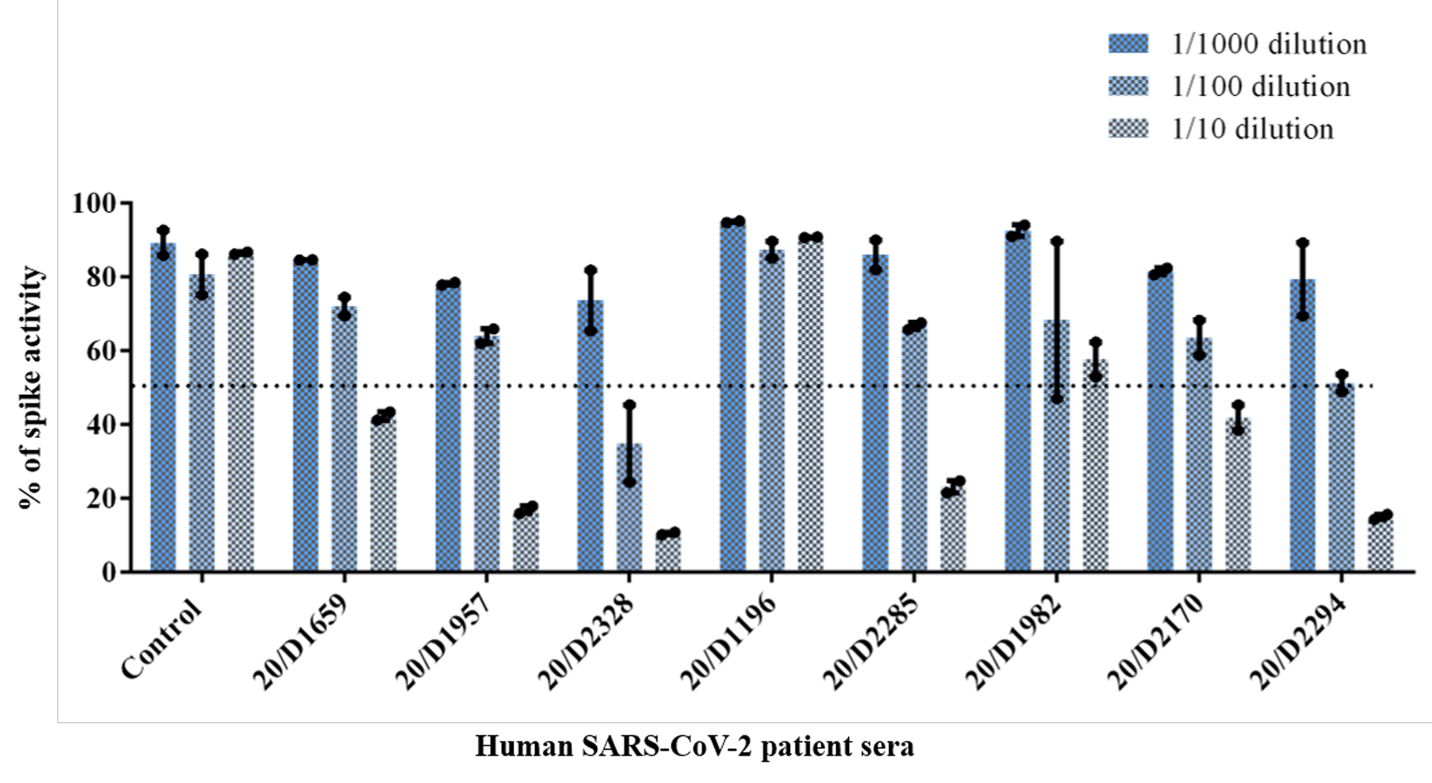


**Figure S20.** Estimation of high affinity antibodies levels in human SARS-CoV-2 patient sera which blocking trimeric spike-hACE2 interaction by RBD-hACE2 competitive ELISA.
